# SGK1, a Serine/Threonine Kinase, Inhibits Prototype Foamy Virus Replication

**DOI:** 10.1128/spectrum.01995-21

**Published:** 2022-04-19

**Authors:** Junshi Zhang, Chunhua Han, Zhenjie Xiong, Manman Qiu, Xiaopeng Tuo, Chenchen Wang, Wentao Qiao, Juan Tan

**Affiliations:** a Key Laboratory of Molecular Microbiology and Technology, Ministry of Education, College of Life Sciences, Nankai University, Tianjin, China; Kumamoto University

**Keywords:** prototype foamy virus, SGK1, Tas, Gag, transcription, protein stability

## Abstract

Foamy viruses (FVs) are complex retroviruses belonging to the *Spumaretrovirinae* subfamily of the *Retroviridae* family. In contrast to human immunodeficiency virus (HIV), another member of the *Retroviridae* family, FVs are nonpathogenic in their natural hosts or in experimentally infected animals. Prototype foamy virus (PFV) is the only foamy virus that can infect humans through cross-species transmission and does not show any pathogenicity after infection. Consequently, PFV is considered a safe and efficient gene transfer vector. Understanding the host proteins involved in the replication of PFV and the mechanism of interaction between the host and the virus might lead to studies to improve the efficiency of gene transfer. To date, only a few host factors have been identified that affect PFV replication. In the present study, we report that PFV infection enhances the promoter activity of *SGK1* (encoding serum/glucocorticoid regulated kinase 1) via the Tas protein signaling pathway, and then upregulates the mRNA and protein levels of SGK1. Overexpression of *SGK1* reduced PFV replication, whereas its depletion using small interfering RNA increased PFV replication. SGK1 inhibits PFV replication by impairing the function of the PFV Tas activation domain in a kinase-independent manner and reducing the stability of the Gag protein in a kinase-dependent manner. In addition, both human and bovine SGK1 proteins inhibit the replication of bovine foamy virus (BFV) and PFV. These findings not only improved our understanding of the function of SGK1 and its relationship with foamy viruses, but also contributed to determining the antiviral mechanism of the host.

**IMPORTANCE** Foamy viruses can integrate into the host chromosome and are nonpathogenic in natural hosts or in experimentally infected animals. Therefore, foamy viruses are considered to be safe and efficient gene transfer vectors. Persistent infection of foamy viruses is partly caused by the restrictive effect of host factors on the virus. However, only a few cellular proteins are known to influence the replication of foamy viruses. In this study, we report that SGK1 inhibits the replication of prototype foamy virus by affecting the function of the transcription activator, Tas, and reducing the stability of the structural protein, Gag. These results will increase our understanding of the interaction between the virus and host factors, deepening our perception of host antiviral defenses and the function of SGK1, and could improve the gene transfer efficiency of foamy viruses.

## INTRODUCTION

Foamy viruses (FVs), also known as spumaretroviruses, are the oldest of the retroviruses, belonging to the *Spumaretrovirinae* subfamily of the *Retroviridae* family (1). FVs can infect humans (2), felines (3), bovines (4), equines (5), and a variety of nonhuman primates (6). FVs induce a strong cytopathic effect after infecting cells *in vitro*, producing characteristic syncytia. However, they are nonpathogenic in naturally or experimentally infected animals, maintaining a long-term persistent infection (7, 8). Prototype foamy virus (PFV) was isolated from a patient with nasopharyngeal carcinoma in Kenya in the 1970s and is the best-characterized member of the FVs (9).

In contrast to most complex retroviruses, foamy viruses have two promoters in their genome. The 5′ long terminal repeat (LTR) promoter regulates the expression of the structural genes, *gag*, *pol*, and *env*. The internal promoter (IP), located near the 3′ end of the *env* gene, regulates the expression of the accessory proteins, Tas and Bet. Tas is a regulatory protein necessary for virus replication, which has two important structural domains. A C-terminal activation domain (AD) (amino acids ~250 to 290) and an N-terminal DNA binding domain (DNA-BD [amino acids ~80 to 210]) (10). Tas regulates FV genes expression by binding to the transactivation responsive elements (TREs) on LTR and IP promoters (11, 12).

FVs cause lifelong persistent infections, which are restricted by a number of host cell factors that inhibit viral replication. A variety of anti-FVs effectors target different steps of the virus replication cycle, including PML nuclear body scaffold (PML), apolipoprotein B mRNA editing enzyme catalytic subunit 3G (APOBEC3G), bone marrow stromal antigen 2 (BST2), interferon induced protein 35 (IFP35), N-myc and STAT interactor (NMI), p53-induced protein with a RING-H2 domain (PIRH2), Schlafen family member 11 (SLFN11), PHD finger protein 11 (PHF11), and TBC1 domain family member 16 (TBC1D16) (13–22). To identify new cell factors that inhibit FVs replication, we determined the differential mRNA expression profiles in PFV-infected cells and found that *SGK1* (encoding serum/glucocorticoid regulated kinase 1) expression was upregulated in PFV-infected cells, suggesting an important role of SGK1 during PFV infection.

SGK1 belongs to the AGC (protein kinase A [PKA]/protein kinase G [PKG]/protein kinase C [PKC]) family of serine/threonine kinases, and is one of the most conserved protein kinases, being present in most eukaryotes (23). SGK consists of three isoforms, SGK1, SGK2, and SGK3, which are highly homologous (24). SGK1 is expressed in all tissues, and its activation depends on the phosphorylation of S422 in the C-terminal domain (25, 26). Activated SGK1 phosphorylates its downstream substrates and regulates ion channels, cell survival, cell proliferation, and cell migration (27–29). SGK1 phosphorylates substrate proteins containing the RXRXX(S/T) motif (30). SGK1 is located in the cytoplasm, nucleus, and plasma membrane (31–35).

SGK1 is a host factor that is implicated in viral infection. SGK1 promotes the replication of certain viruses. For example, SGK1 stimulates the nuclear export of the ribonucleoprotein of influenza A virus (IAV) or facilitates the early steps of influenza virus replication (36, 37). SGK1 facilitates the entry of human immunodeficiency virus type 1 (HIV-1), and *in vitro*, HIV-1 Tat peptides inhibit SGK1 via substrate competition (38, 39). SGK1 also stimulates the productive infection of bovine herpesvirus 1 (BoHV-1) and herpes simplex virus 1 (HSV-1) (40, 41). Contrastingly, SGK1 plays an inhibitory role in the replication of certain viruses. For example, SGK1 can reduce hepatitis C virus (HCV) production (42). Furthermore, the expression of SGK1 is also regulated by the virus. The SGK1 protein levels increased during coxsackievirus B3 (CVB3) and BoHV-1 infection (40, 43), while HCV infection decreases the mRNA and protein levels of SGK1 (42). However, whether it promotes or inhibits viruses, the molecular mechanisms of these effects remain insufficiently studied. In the present study, we aimed to investigate the effects of SGK1 on the PFV replication.

## RESULTS

### Upregulation of SGK1 by PFV infection.

**FIG 1:**
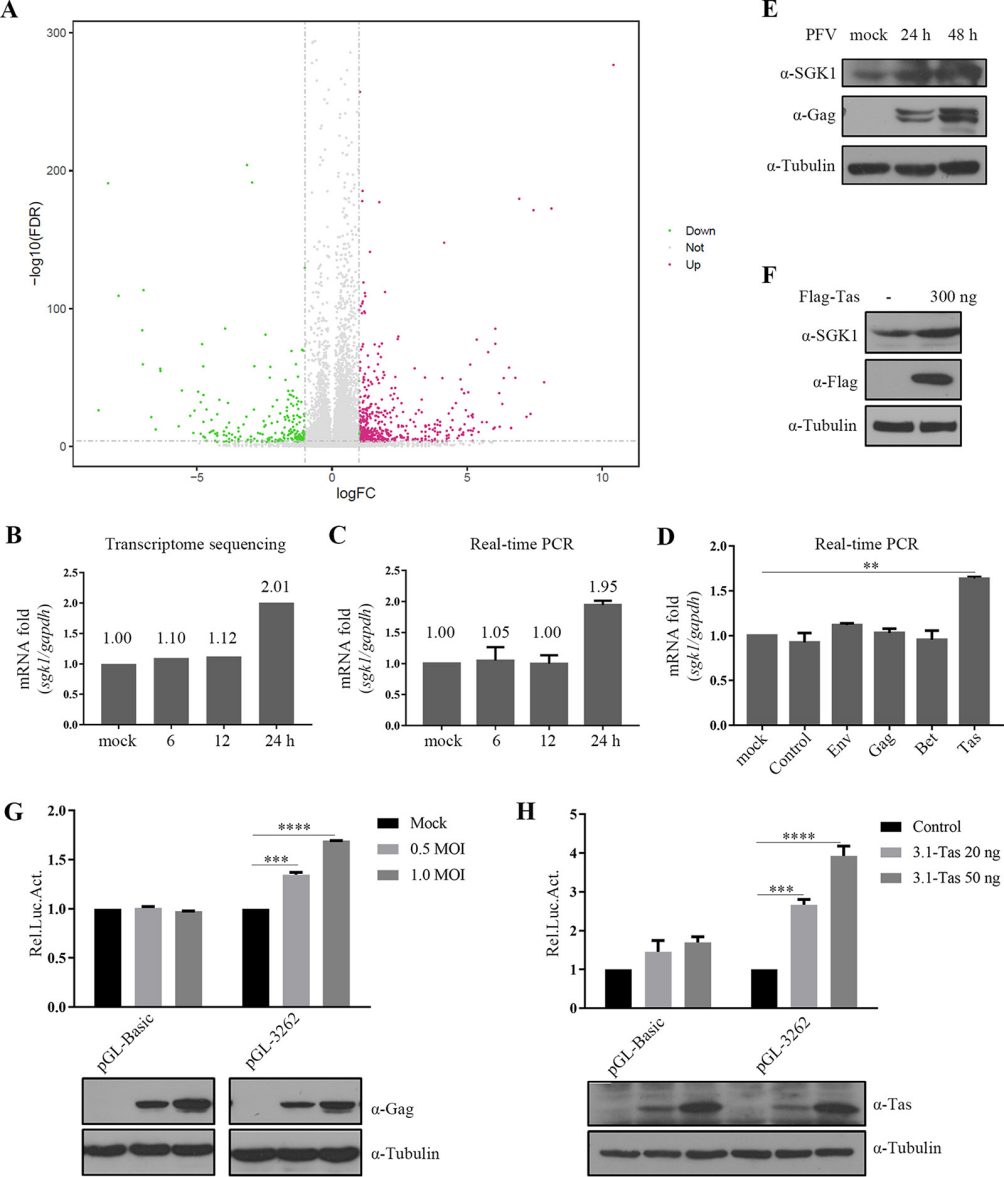
Upregulation of SGK1 by PFV infection. (A) Basic information from RNA-seq. Volcano plots of significant differential expressed mRNAs between cell infected with PFV (MOI = 1.2) for 24 h and mock infected cells. The *x* axis represents the log_2_ fold change, and the *y* axis represents -log_10_ (false discovery rate (FDR). (B) The result of transcriptomic sequencing. (C) PFV (MOI = 1.0) infected HT1080 cells at 6 h, 12 h, and 24 h. Total RNA was extracted and reverse transcribed into cDNA. Real-time PCR was performed using gene-specific primers to detect the mRNA level of *SGK1*. (D) HT1080 cells (1 × 10^5^) were transfected with Env, Gag, Bet, Tas, and empty vector (300 ng). After 24 h of transfection, total RNA was extracted and reverse transcribed into cDNA. Real-time PCR was performed using gene-specific primers to detect the mRNA level of *SGK1*. (E) HT1080 cells (1 × 10^5^) were infected with PFV (MOI = 1.0) for 24 h and 48 h, the cells were collected and the level of endogenous SGK1 was detected by anti-SGK1 antibody. (F) HT1080 cells (1 × 10^5^) were transfected with Flag-Tas, and after 48 h, cells were collected and the level of endogenous SGK1 was detected using Western blotting. (G) HT1080 cells (1 × 10^5^) were transfected with pGL-*SGK1* P3262 (50 ng) or empty vector, and at 24 h posttransfection, they were infected with PFV with a gradient of MOI for 24 h, and luciferase activities were measured and corrected by β-gal catalytic activities. (H) HEK293T cells (2 × 10^5^) were transfected with pGL-*SGK1* P3262 (5 ng), combined with 3.1-Tas 20 ng, 50 ng, and empty vector, pCMV-β-gal (25 ng) was transfected to normalize the transfection efficiency. At 48 h posttransfection, luciferase activities were measured and corrected by β-gal catalytic activities. Data are expressed as the means ± standard deviations. Data are representative of two or three independent experiments. One-way ANOVA was used to perform the statistical test. **, *P < *0.01; ***, *P < *0.001; ****, *P < *0.0001.

To screen cellular factors involved in PFV replication, we performed transcriptome sequencing on the cellular RNA of PFV-infected HT1080 cells at 6 h, 12 h, and 24 h. A volcano plot (Fig. 1A) showed that compared with the mock-infected group, PFV infection significantly changed the mRNA levels of certain genes 24 h later, among which the mRNA levels of 294 genes were upregulated and 100 genes were downregulated. These 394 differentially expressed genes (DEGs) were functionally annotated using gene ontology (GO) annotation and Kyoto Encyclopedia of Genes and Genomes (KEGG) pathway enrichment analyses. GO analysis showed that DEGs are highly enriched in ossification, nucleoside−triphosphatase regulator activity, GTPase regulator activity, and GTPase activator activity (Fig. S1A). The breast cancer pathway and mammalian target of rapamycin (mTOR) signaling pathway were the most correlated pathways in the KEGG analysis (Fig. S1B). The mTOR pathway is involved in the infection of many DNA and RNA viruses and plays an important role in viral replication (44). DEGs in the mTOR signaling pathway were shown in Fig. S1C. Among them, we noted that SGK1 was reported to affect the replication of certain viruses; however, the exact mechanisms involved are poorly understood. Therefore, we decided to focus on SGK1 in the present study.

To explore SGK1’s involvement in PFV replication, we first verified the results of transcriptome sequencing. The quantitative real-time PCR (qPCR) results showed that compared with the uninfected group, the endogenous *SGK1* mRNA level was significantly upregulated at 24 h after PFV infection (Fig. 1C), which was consistent with the results of transcriptome sequencing (Fig. 1B). Overexpression of PFV Tas increased the level of *SGK1* mRNA (Fig. 1D). Additionally, PFV infection (Fig. 1E) or transfection with the Tas plasmid (Fig. 1F) increased the SGK1 protein level. Next, we explored whether PFV infection affects the promoter activity of *SGK1*. PFV infection (Fig. 1G) or transfection with the Tas plasmid (Fig. 1H) upregulated the *SGK1* promoter activity in a dose-dependent manner. Thus, PFV infection enhances the promoter activity of *SGK1* via the Tas protein, resulting in upregulated mRNA and protein levels of endogenous SGK1.

### Overexpression of SGK1 inhibits PFV replication.

We next studied the effect of SGK1 on PFV replication. We used a PFV indicator cell line (PFVL) to measure virus titers. PFVL was created by transfecting baby hamster kidney-21 (BHK-21) cells with a reporter plasmid containing a firefly luciferase gene driven by the PFV LTR promoter, such that luciferase expression depends on the PFV transactivator Tas, and the expression level is directly proportional to the amount of Tas. Therefore, PFVL can be used to indicate the PFV titer and is more sensitive than TCID50 (the median tissue culture infectious dose) (45). In this PFV-activated luciferase assay, HEK293T cells were transfected with the *SGK1* overexpressing plasmid and the PFV full-length infectious clone, pcPFV. After 48 h, the culture supernatant (cell-free PFV particles) and transfected cells (cell-associated PFV particles) were harvested and incubated with PFVL. Forty-eight hours later, the luciferase activity was detected to indicate the virus titer. Western blotting was used to detect the levels of viral proteins in the transfected HEK293T cells. The levels of both cell-free and cell-associated PFV were reduced by *SGK1* overexpression. Consistently, *SGK1* overexpression significantly reduced the expression levels of Gag, Bet, and Tas in transfected HEK293T cells compared with those in control cells (Fig. S2A). Similar results were observed using HT1080 cells (Fig. S2B). Thus, *SGK1* overexpression inhibits PFV replication.

**FIG 2:**
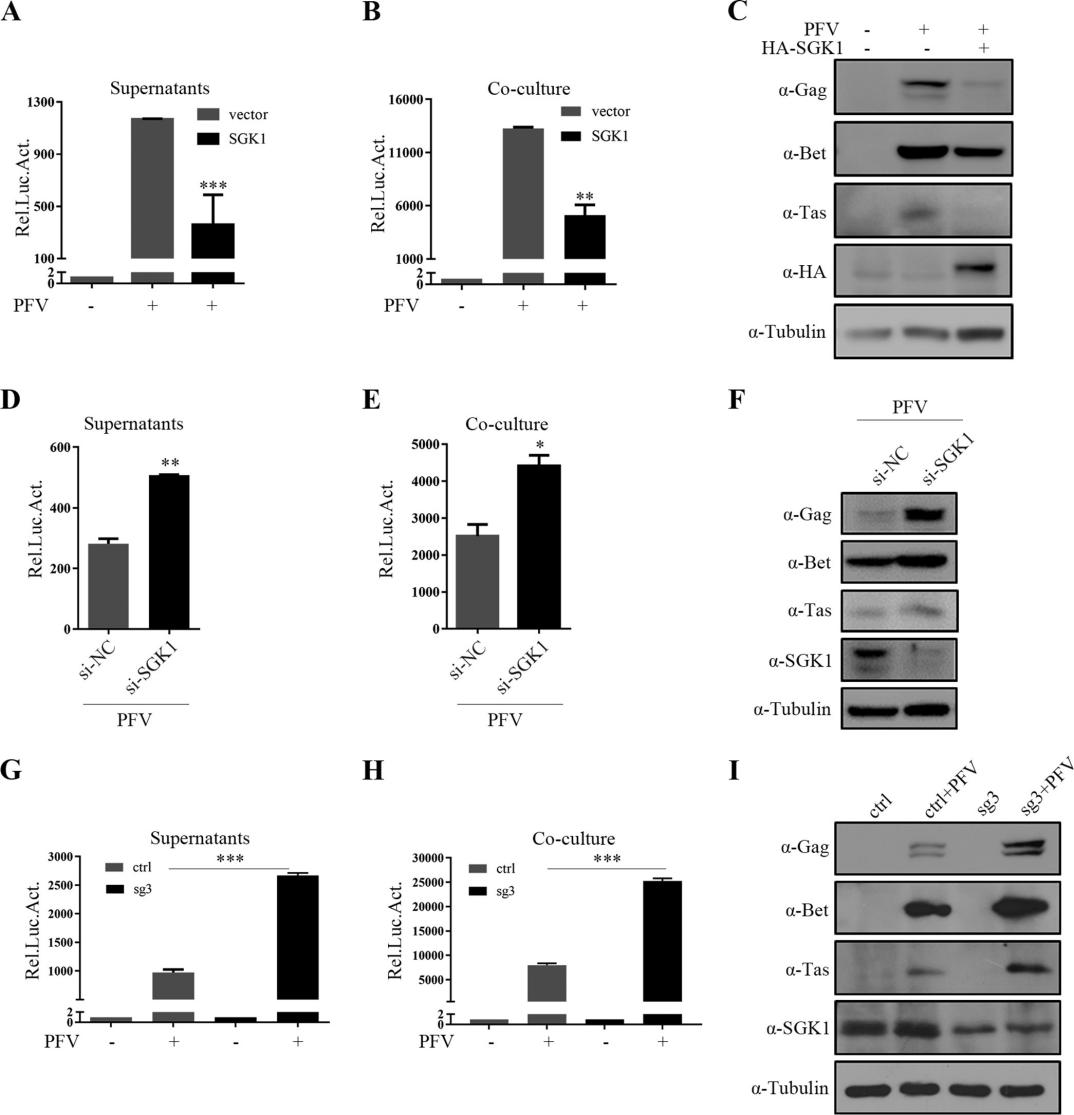
SGK1 inhibits PFV replication. (A to C) HT1080-Control and HT1080 stable expression cells (1 × 10^5^) were infected with PFV (MOI = 0.6). At 48 h postinfection, 600 μL of the supernatants (A) or 1/10 infected HT1080 cell lines (B) were incubated with PFVL cells (1 × 10^5^), the luciferase activity was measured 48 h later, and the rest of the infected cells were lysed for Western blotting (C). (D to F) HT1080-siControl and HT1080-siSGK1 cells (1 × 10^5^) were infected with PFV (MOI = 0.6). At 24 h postinfection, 600 μL of the supernatants (D) or 1/10 infected HT1080 cells (E) were incubated with PFVL cells (1 × 10^5^), and the luciferase activity was measured 48 h later. (F) The rest of the infected cells were lysed for Western blotting. (G to I) PFV (MOI = 0.6) infected Control and sg3 cell lines (1 × 10^5^). At 48 h postinfection, 600 μL of the supernatants (G) or 1/10 infected HT1080 cells (H) were incubated with PFVL cells (1 × 10^5^), and the luciferase activity was measured 48 h later. (I) The remaining infected cells were lysed for Western blotting. Data are expressed as the means ± standard deviations. Data are representative of three independent experiments. One-way ANOVA was used to perform the statistical test. *, *P < *0.05; **, *P < *0.01; ***, *P < *0.001.

To further confirm the inhibitory effect of SGK1 on PFV replication, we used a retroviral vector system to screen cell lines stably overexpressing *SGK1* and control cell lines. After PFV infection, the luciferase activity and Western blotting were used to indicate the viral replication and viral protein levels in the cells. The infectivity of PFV in HT1080 cells stably overexpressing *SGK1* was significantly lower than that in the control cells (Fig. 2A to C), further indicating that SGK1 inhibits the replication of PFV.

### Knockdown of endogenous *SGK1* enhances PFV replication.

Next, we explored whether endogenous SGK1 can also inhibit PFV replication. First, we detected endogenous SGK1 levels in HeLa, HT1080, and HEK293T cells. The results showed the highest level of SGK1 in HT1080 cells (Fig. S3A); therefore, HT1080 cells were chosen to knock down endogenous *SGK1* expression. We designed small interfering RNAs (siRNAs) to knock down endogenous *SGK1*. After detecting the knockdown efficiency using real-time-PCR (Fig. S3B), siRNA #2 was selected for the virus infection experiment. The knockdown of endogenous *SGK1* enhanced PFV infectivity (Fig. 2D–F). In addition, we used the clustered regularly interspaced short palindromic repeats (CRISPR)-CRISPR-associated protein 9 (Cas9) system to create *SGK1* knockdown cell lines, and transfected pcPFV into three *SGK1* knockdown cell lines and control cell lines. Compared with the control cells, knockdown of endogenous *SGK1* enhanced PFV replication (Fig. S3C to E), and the knockdown effect was more apparent in cell line #3. We selected knockdown cell line #3 for sequencing and analyzed the knockdown efficiency. The results showed obvious multisignal peaks starting around the cleavage site of single guide (sg) sg3 in the knockdown cells (Fig. S3F), and TIDE (tracking indels by decomposition) analysis indicated an overall knockdown efficiency of 81.6% (Fig. S3G). We used this cell line for PFV infection experiments and found that *SGK1* knockdown increased the infectivity of PFV (Fig. 2G to I). These results suggest that endogenous SGK1 also inhibits PFV replication.

### Non-human SGK1 inhibits PFV replication.

**FIG 3:**
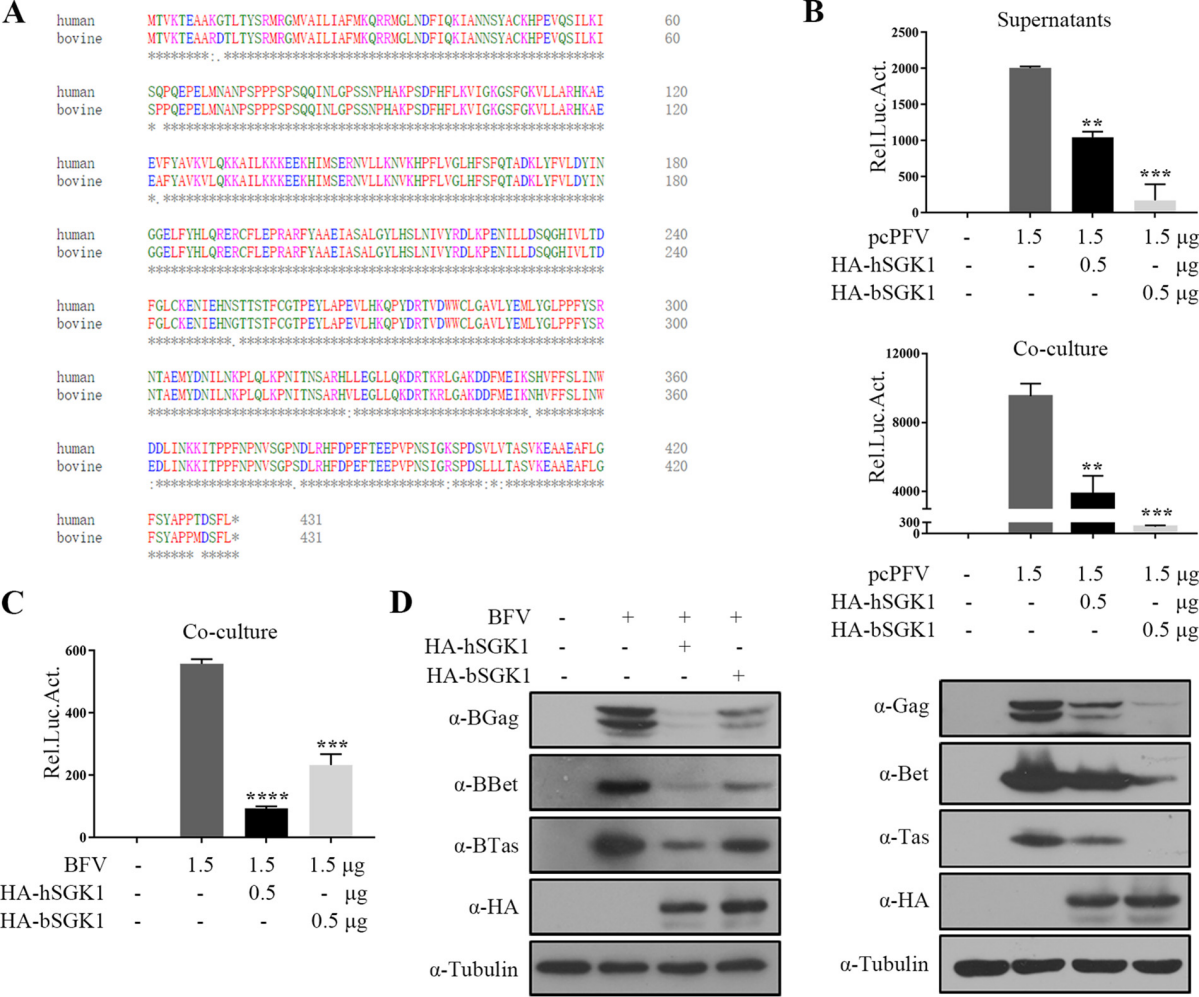
Non-human SGK1 inhibits PFV replication. (A) Homology of human and bovine SGK1 proteins. Asterisks (*) denote identical amino acids. (B) HEK293T cells (2 × 10^5^) were co-transfected with pcPFV (1.5 μg) and empty vector, hSGK1 or bSGK1 (0.5 μg). At 48 h posttransfection, 600 μL of the supernatants or 1/20 transfected cells were incubated with PFVL cells (1 × 10^5^), the luciferase activity was measured 48 h later, and the rest of transfected cells were lysed for Western blotting. (C, D) HEK293T cells (2 × 10^5^) were co-transfected with BFV (1.5 μg) and empty vector, hSGK1 or bSGK1 (0.5 μg). At 48 h posttransfection, 1/20 transfected cells were incubated with BFVL cells (1 × 10^5^), the luciferase activity was measured 48 h later (C). (D) The remaining transfected cells were lysed for Western blotting. Data are expressed as the means ± standard deviations. Data are representative of two independent experiments. One-way ANOVA was used to perform the statistical test. **, *P < *0.01; ***, *P < *0.001; ****, *P < *0.0001.

Many host restriction factors can inhibit virus replication across species. Given that human SGK1 (hSGK1) shares 97% sequence similarity with bovine SGK1 (bSGK1) (Fig. 3A), we explored whether bSGK1 affects PFV replication. bSGK1 and hSGK1 expression constructs were co-transfected with pcPFV into HEK293T cells, separately. Interestingly, bSGK1 decreased the infectivity of PFV in the supernatants and cells (Fig. 3B) to a greater the extent of than that of hSGK1.

We next examined the effect of hSGK1 on bovine foamy virus (BFV) replication. The infectious clone pBS-BFV was co-transfected into HEK293T cells with hSGK1 and bSGK1 expression constructs, separately. BFV can only establish infection through cell-to-cell transmission; therefore, we harvested the cells at 48 h after transfection and incubated them with the BFVL indicator cell line. The BFV titer was determined by luciferase assays and viral protein levels in the transfected cells was detected using Western blotting. hSGK1 decreased the infection of BFV through cell-to-cell contact (Fig. 3C) to a greater extent than that of bSGK1. The cellular BFV protein levels also decreased (Fig. 3D).

### SGK1 inhibits Tas from transactivating PFV LTR and IP promoters.

**FIG 4:**
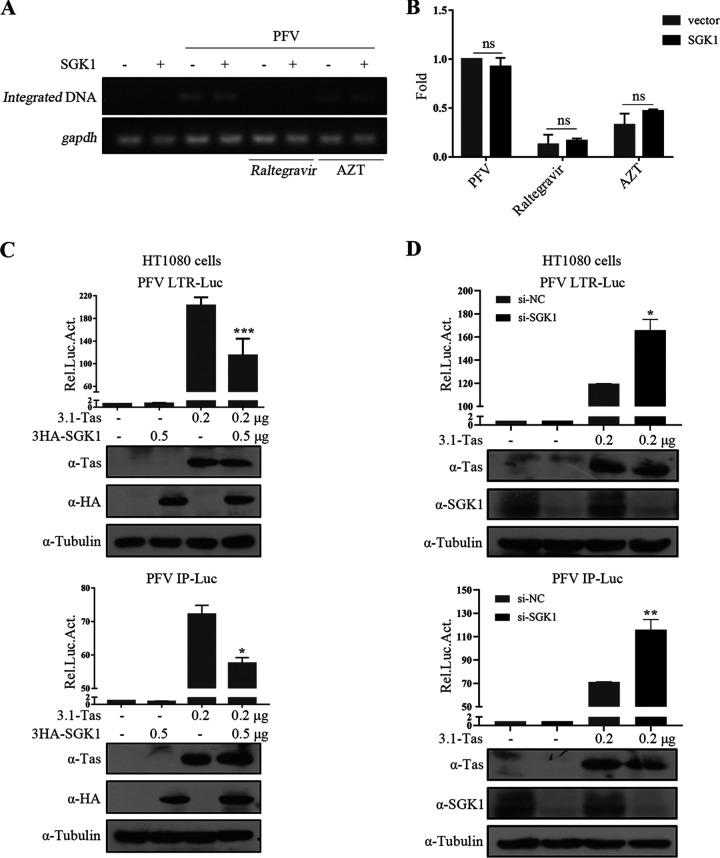
SGK1 inhibits Tas from transactivating PFV LTR and IP promoters. Levels of integrated proviral DNA were measured using semiquantitative PCR (A) and real-time PCR (B). (C) HT1080 cells (1 × 10^5^) were transfected with LTR-Luc (0.05 μg) or IP-Luc (0.025 μg), combined with 3.1-Tas and empty vector or SGK1. At the same time, pCMV-β-gal (0.05 μg) was transfected to normalize the transfection efficiency. At 48 h posttransfection, luciferase activities were measured and corrected by β-gal catalytic activities. The remaining cell lysates were used for Western blotting. (D) HT1080-siControl and HT1080-siSGK1 cells (1 × 10^5^) were transfected with LTR-Luc (0.05 μg) or IP-Luc (0.025 μg) and 3.1-Tas. At the same time, pCMV-β-gal (0.05 μg) was transfected to normalize transfection efficiency. At 48 h posttransfection, luciferase activities were measured and corrected by β-gal catalytic activities. The remaining cell lysates were used for Western blotting. Data are expressed as the means ± standard deviations. Data are representative of three independent experiments. One-way ANOVA was used to perform the statistical test. *, *P < *0.05; **, *P < *0.01; ***, *P < *0.001; and ns for *P > *0.05.

Additional studies were conducted to further investigate the mechanism by which SGK1 inhibits PFV replication. The replication cycle of retroviruses is artificially divided into an early and late stages based on integration as a stage dividing line. To determine which stage of the PFV life cycle is inhibited by SGK1, we tested whether SGK1 inhibits PFV integration. Based on the principle of Alu-PCR, the integrated PFV genome was detected using semi-quantitative PCR (Fig. 4A) and real-time PCR (Fig. 4B), respectively, which showed that *SGK1* overexpression had no significant effect on PFV integration.

The above results suggested that SGK1 might exert its antiviral function in the late stage of PFV replication; therefore, we tested whether SGK1 affected the transcription of PFV. Tas binds to the PFV LTR and IP promoters and activates viral transcription, playing an important role in the virus life cycle (11, 46). Therefore, whether SGK1 affected Tas activation of LTR and IP promoters was examined. The results showed that SGK1 had no significant effect on the basic activity of the LTR and IP promoters (Fig. 4C); however, SGK1 inhibited the ability of Tas to activate the LTR and IP promoters. Similar results were obtained in HEK293T and HeLa cells (Fig. S4A, B). Next, the effect of endogenous SGK1 on Tas transactivation was explored. Endogenous *SGK1* knockdown enhanced Tas-mediated transactivation of the LTR and IP promoters (Fig. 4D). These results suggested that SGK1 inhibits Tas transactivating of the PFV LTR and IP promoters.

### SGK1 inhibits the function of the Tas activation domain.

**FIG 5:**
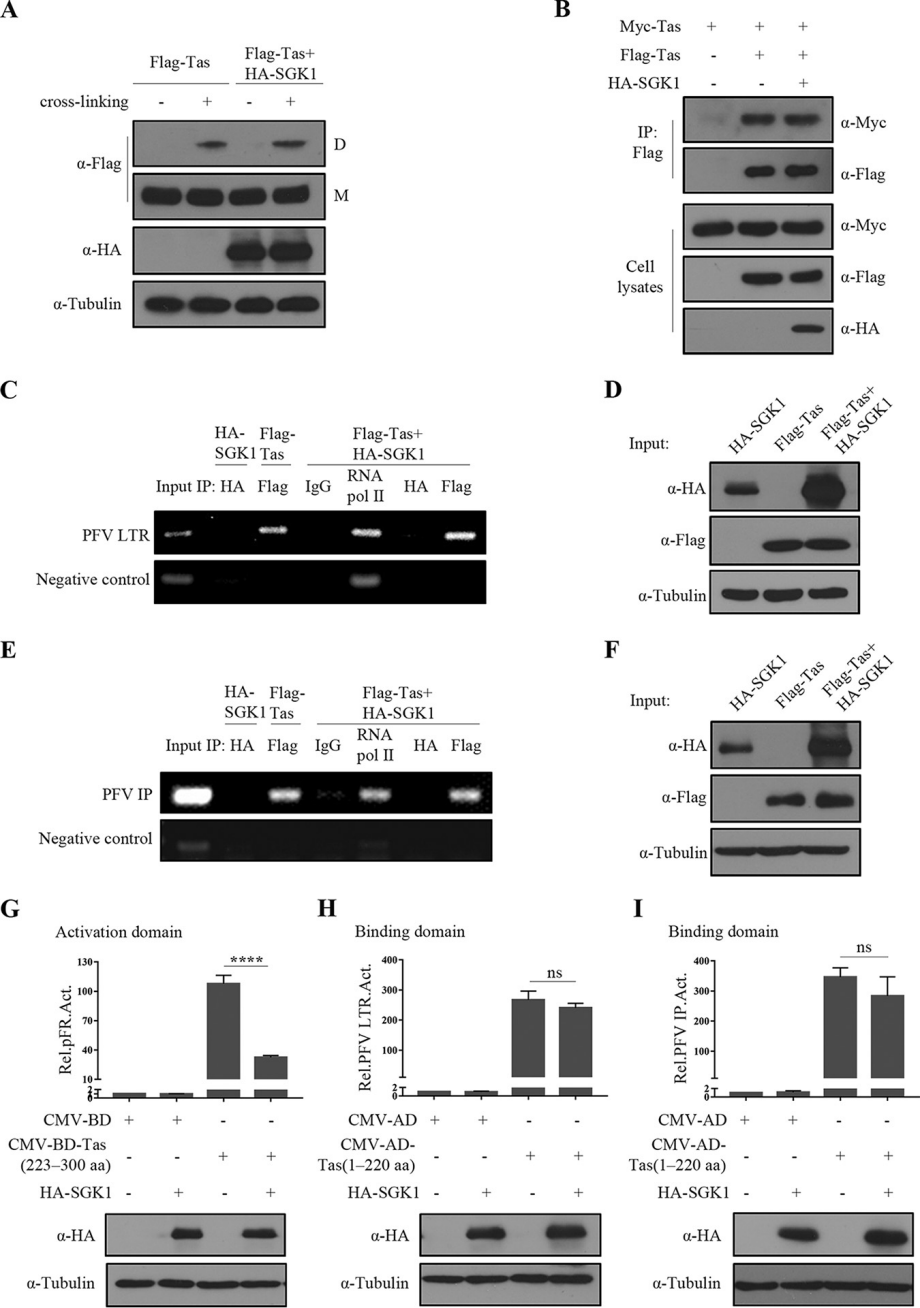
SGK1 inhibits the function of Tas activation domain. (A) HEK293T cells (5 × 10^5^) were transfected with Flag-Tas (1 μg) and empty vector or HA-SGK1 (1 μg). At 48 h posttransfection, cells were incubated with or without the cross-linking chemical. Cell lysates were analyzed by Western blotting. The positions of Flag-Tas monomers (M) and dimmer (D) are indicated. (B) HEK293T cells (4 × 10^6^) were transfected with Myc-Tas (3 μg), Flag-Tas (3 μg), and empty vector or HA-SGK1 (3 μg). At 48 h posttransfection, co-immunoprecipitation was performed using Flag antibodies. Western blotting of samples from cell lysates and immunoprecipitates using Myc, Flag, and HA antibodies. (C) PFVL cells (9 × 10^5^) were co-transfected with HA-SGK1 (5 μg), Flag-Tas (5 μg), Flag-Tas (5 μg) + HA-SGK1 (5 μg), respectively. At 48 h posttransfection, cells were subjected to a ChIP assay as described in Materials and Methods. Subsequently, PCR amplification was carried out to detect LTR in the immunoprecipitated chromatin fragments. (D) Part of the cell lysates was used to detect protein levels using Western blotting. (E) HEK293T cells (2.5 × 10^6^) were co-transfected with PFV IP-Luc (3 μg) and HA-SGK1 (5 μg), Flag-Tas (5 μg), Flag-Tas (5 μg) + HA-SGK1 (5 μg), respectively. At 48 h posttransfection, cells were subjected to a ChIP assay. Subsequently, PCR amplification assay was carried out to detect IP in the immunoprecipitated chromatin fragments. (F) Part of the cell lysates was used to detect protein levels using Western blotting. (G) HEK293T cells (2 × 10^5^) were transfected with pCMV-BD or pCMV-BD-Tas (223 to 300 aa) (0.02 μg), HA-SGK1 or empty vector (0.3 μg), pFR-Luc (0.01 μg) and pCMV-β-gal (0.025 μg). After 48 h of transfection, luciferase activity was measured. (H, I) HEK293T cells (2 × 10^5^) were transfected with pCMV-AD or pCMV-AD-Tas (1 to 220 aa) (0.3 μg), HA-SGK1 or empty vector (0.3 μg), LTR-Luc (0.05 μg) (H) or IP-Luc (0.025 μg) (I), and pCMV-β-gal (0.025 μg). After 48 h of transfection, luciferase activity was measured. Data are expressed as the means ± standard deviations. Data are representative of three independent experiments. One-way ANOVA was used to perform the statistical test. ****, *P < *0.0001; ns for *P > *0.05.

Tas forms multimeric complexes in mammalian cell nuclei, although their function remains unknown (47, 48). Moreover, Tas binds directly to the PFV LTR or IP promoters through its N-terminal DNA binding domain (amino acids ~ 80 to 210), and activates transcription through its C-terminal activation domain (amino acids ~ 250 to 290) (10). The DNA binding domain and activation domain can be functionally separated. Fusion of the DNA binding domain to a heterologous activation domain can activate the expression of PFV LTR-Luc or IP-Luc, while fusion of the activation domain to a heterologous DNA binding domain (such as yeast GAL4) can activate transcription in mammal and yeast cells (49). Next, SGK1’s effects on Tas multimer formation, DNA binding, and activation were studied. First, we found that SGK1 did not affect Tas multimer formation (Fig. 5A) and Tas-Tas protein interactions (Fig. 5B).

Tas must bind to promoters to activate transcription after nuclear entry; therefore, chromatin immunoprecipitation (CHIP) was used to detect the effect of SGK1 on Tas DNA binding. According to the results of semi-quantitative PCR, Tas bound to the LTR promoter, which was not affected by co-transfection of the *SGK1* overexpression vector (Fig. 5C). The protein levels of SGK1 and Tas were detected using Western blotting (Fig. 5D). In addition, SGK1 did not affect Tas binding to the PFV IP promoter (Fig. 5E and F). Thus, SGK1 does not affect the DNA binding ability of Tas. A mammalian two-hybrid system was used to further explore the effect of SGK1 on Tas DNA binding and transcription activation. We constructed a plasmid (pCMV-AD-Tas 1 to 220 aa) that fused the Tas DNA binding domain to a heterologous transactivator domain to test whether Tas DNA binding was inhibited by SGK1. In addition, a plasmid (pCMV-BD-Tas 223 to 300 aa) that fused the Tas activation domain to a heterologous DNA binding domain was used to test whether Tas transcription activation was inhibited by SGK1. The results showed that SGK1 affected the function of the Tas activation domain (Fig. 5G) but did not affect the function of the Tas DNA binding domain (Fig. 5H and I), similar to the CHIP results. These results indicated that SGK1 affects the Tas transcription activation domain and thus regulates Tas transactivation of the PFV LTR and IP promoters.

### SGK1 interacts with the Tas activation domain.

**FIG 6:**
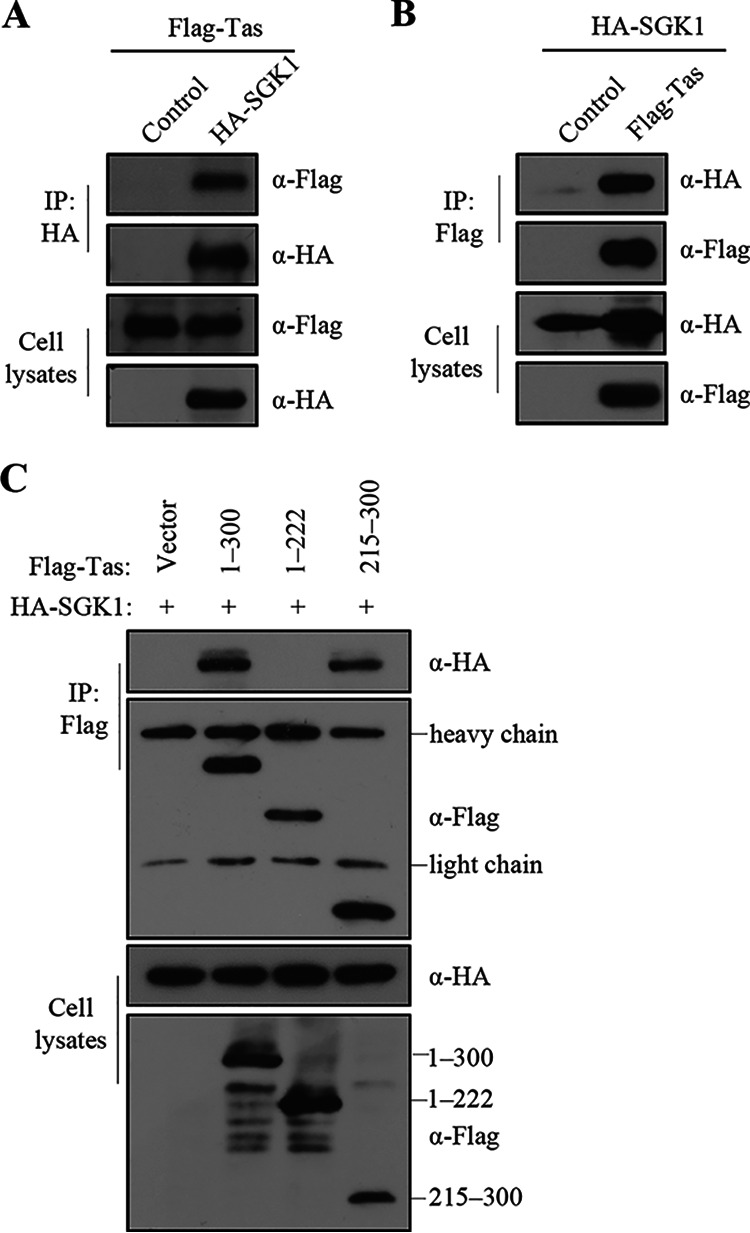
SGK1 interacts with Tas activation domain. (A) HEK293T cells (4 × 10^6^) were transfected with Flag-Tas (3 μg) and empty vector or HA-SGK1 (3 μg). At 48 h posttransfection, co-immunoprecipitation was performed with HA antibodies. Western blotting of samples from cell lysates and immunoprecipitates using HA and Flag antibodies. (B) HEK293T cells (4 × 10^6^) were transfected with HA-SGK1 (3 μg) and empty vector or Flag-Tas (3 μg). At 48 h posttransfection, co-immunoprecipitation was performed with Flag antibodies. Western blotting of samples from cell lysates and immunoprecipitates using HA and Flag antibodies. (C) HA-SGK1 (3 μg) was transiently transfected into HEK293T cells (4 × 10^6^) together with the indicated wild-type or truncated Tas DNA (3 μg). At 48 h posttransfection, co-immunoprecipitation was performed using Flag antibodies. Western blotting of samples from cell lysates and immunoprecipitates using HA and Flag antibodies.

Next, investigated the interaction between SGK1 and Tas using co-immunoprecipitation (Co-IP), which confirmed that SGK1 interacted with Tas in cells (Fig. 6A and B). To determine the mechanism by which SGK1 affects the function of the Tas activation domain, we co-transfected HEK293T cells with the SGK1 construct, the empty vector, the Tas construct, and constructs expressing a Tas deletion mutant (215 to 300 aa), which contained the activation domain or a Tas deletion mutant (1 to 222 aa) that contained the DNA binding domain, respectively. The Co-IP results showed that SGK1 interacted with Tas and the mutant retaining the Tas activation domain, but not with the mutant retaining the Tas DNA binding domain (Fig. 6C). These results implied that SGK1 interacts with the Tas activation domain to affect Tas-mediated transcriptional activation in cells.

### SGK1 inhibits the transactivation function of Tas in a kinase-independent manner.

**FIG 7:**
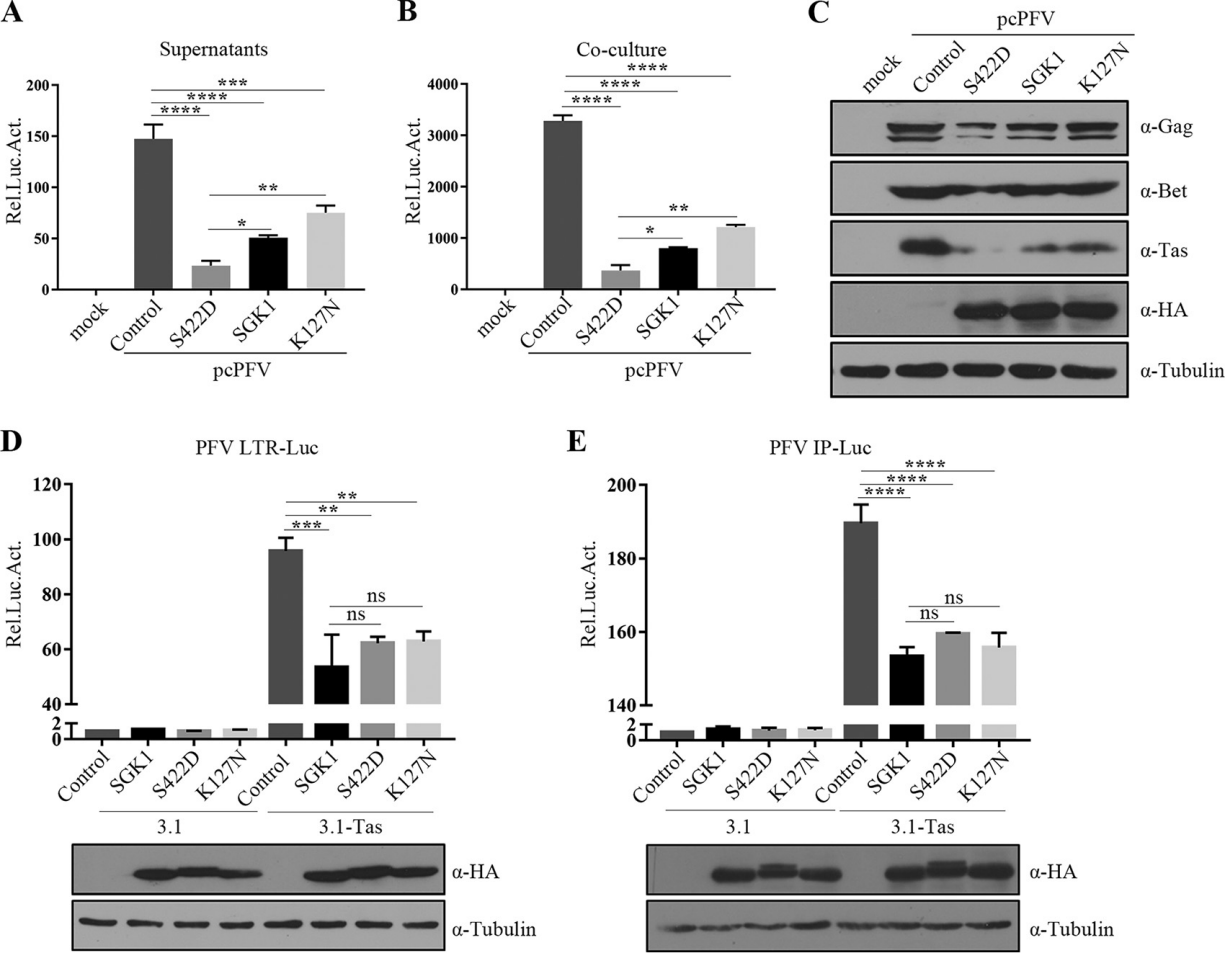
SGK1 inhibits the transactivation function of Tas in a kinase-independent manner. (A to C) HEK293T cells (2 × 10^5^) were co-transfected with pcPFV (0.5 μg) and empty vector, S422D, SGK1, or K127N (0.5 μg). At 48 h posttransfection, 600 μL of the supernatants (A) or 1/20 transfected cells (B) were incubated with PFVL cells (1 × 10^5^), the luciferase activity was measured 48 h later. (C) The rest of transfected cells were lysed for Western blotting. (D, E) HEK293T cells (2 × 10^5^) were transfected with LTR-Luc (0.025 μg) (D) or IP-Luc (0.01 μg) (E), combined with 3.1-Tas (0.1 μg) and empty vector or SGK1, SGK1-S422D, and SGK1-K127N (0.3 μg). At the same time, pCMV-β-gal (0.025 μg) was transfected to normalize the transfection efficiency. At 48 h posttransfection, luciferase activities were measured and corrected by β-gal catalytic activities. Remaining cell lysates were used for Western blotting. Data are expressed as the means ± standard deviations. Data are representative of three independent experiments. One-way ANOVA was used to perform the statistical test. *, *P < *0.05; **, *P < *0.01; ***, *P < *0.001; ****, *P < *0.0001; and ns for *P > *0.05.

Although SGK1 is a serine/threonine kinase, the exact role of enzymatically active SGK is unclear (50). To determine whether the kinase activity of SGK1 plays a role in its inhibition of PFV replication, we used a constitutively kinase active form of SGK1 (SGK1-S422D) (51), and a dominant negative, kinase-deficient SGK1 mutant (SGK1-K127N) (52). The results showed that SGK1-S422D had the strongest inhibitory effect on PFV, followed by SGK1, and SGK1-K127N had the weakest inhibitory effect, but could still inhibit PFV replication (Fig. 7A to C). Thus, SGK1’s kinase activity might be involved PFV replication inhibition.

We further explored the kinase-dependent effect of SGK1 on Tas transactivation of PFV LTR and IP promoters, and found that both SGK1-S422D and SGK1-K127N could inhibit Tas transactivation of the PFV LTR (Fig. 7D) and IP (Fig. 7E) promoters to the same extent as the wild type. These results suggested that SGK1 inhibits Tas transactivation of the PFV LTR and IP promoters in a kinase-independent manner.

### SGK1 affects the stability of the PFV Gag protein in a kinase-dependent manner.

**FIG 8:**
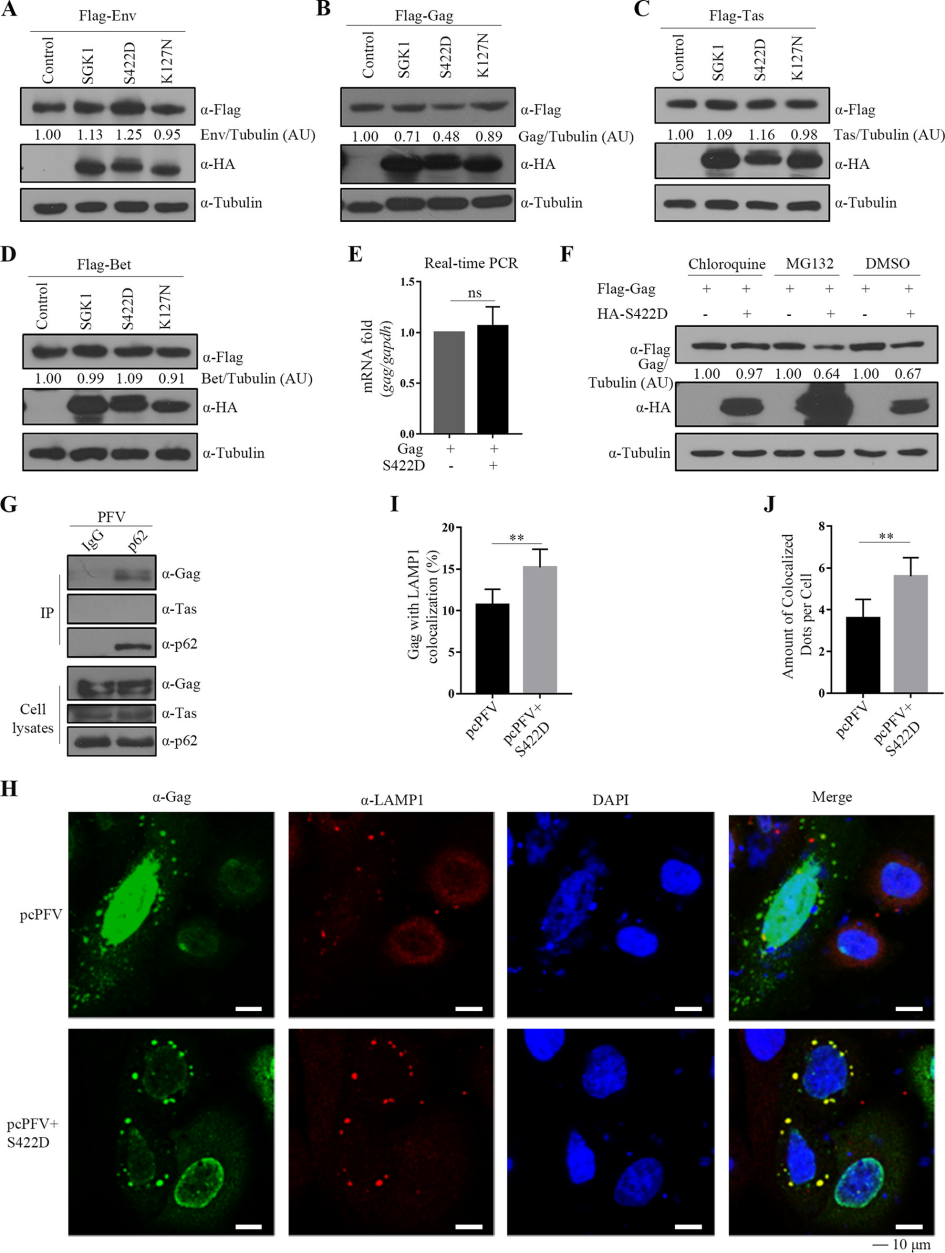
SGK1 affects the stability of PFV Gag protein in a kinase-dependent manner. (A to D) HEK293T cells (2 × 10^5^) were transfected with Flag-Env (0.3 μg) (A), Flag-Gag (0.6 μg) (B), Flag-Tas (0.6 μg) (C), Flag-Bet (0.6 μg) (D), combined with SGK1, SGK1-S422D, SGK1-K127N, or empty vector (0.3 μg). At 48 h posttransfection, cells were harvested for Western blotting analysis. (E) HEK293T cells (5 × 10^5^) were co-transfected with Flag-Gag (0.6 μg) and SGK1-S422D or empty vector (0.3 μg), at 48 h posttransfection, the cells were harvested and RNA was extracted, and then reverse transcribed into cDNA for real-time PCR detection. (F) HEK293T cells (2 × 10^5^) were co-transfected with Flag-Gag (0.6 μg) and SGK1-S422D or empty vector (0.3 μg), and then cells were treated with DMSO, MG132 (20 μM), Chloroquine (100 μM) for 6 h, respectively. Protein expression was analyzed using Western blotting. Protein levels were quantified by densitometry and normalized to Tubulin levels. AU, arbitrary units. (G) HT1080 cells (2 × 10^6^) were infected with PFV stock (MOI = 1.0), at 48 h postinfection, co-immunoprecipitation was performed with IgG and p62 antibodies. Western blotting of samples from cell lysates and immunoprecipitates using Gag, Tas, and p62 antibodies. (H) HeLa cells (3 × 10^4^) were transfected with pcPFV (0.5 μg) and S422D or empty vector (0.5 μg). At 48 h posttransfection, an indirect immunofluorescence assay was used to localize Gag (with Gag antibody and fluorescein isothiocyanate [FITC]-conjugated secondary antibody) and LAMP1 (with LAMP1 antibody and tetramethyl rhodamine isocyanate [TRITC]-conjugated secondary antibody). Nuclei were visualized via 4′,6-diamidino-2-phenylindole (DAPI) staining. Representative images are shown. Scale bars, 10 μm. (I, J) Quantitation of the data in Fig. 8H. (I) The percent of cells in which Gag colocalized with LAMP1. (J) Amount of colocalized dots per cell. Graphs show the means ± the SEM; seven random fields and 20 cells per field were examined by confocal microscopy. **, *P* < 0.01; ns for *P* > 0.05.

SGK1-S422D had the strongest inhibitory effect on PFV replication, with equivalent inhibitory effects on transcription to SGK1 and K127N. SGK1 can affect the stability of some cellular proteins in a kinase-dependent manner (52–54). Therefore, we next explored the effects of different active forms of SGK1 on the levels of PFV proteins. The results showed that SGK1-S422D affected the level of the PFV Gag protein, but not the levels Env, Tas, and Bet (Fig. 8A to D). The real-time PCR showed that SGK1-S422D did not affect the mRNA levels of Gag (Fig. 8E).

To determine whether the degradation of Gag proteins by SGK1-S422D is mediated by the proteasomal or lysosomal pathways, we used proteasomal and lysosomal inhibitors. SGK1-S422D-induced Gag degradation could be rescued by the lysosome inhibitor, chloroquine, but not by the proteasome inhibitor (Fig. 8F). For confirmation, siRNAs targeting *ATG5* (encoding autophagy related 5) were used to inhibit autophagy. Transfection of siATG5 in HEK293T cells significantly reduced the ATG5 mRNA and protein levels (Fig. S5A, B). Indeed, autophagy inhibition by siATG5 prevented SGK1-S422D-mediated Gag degradation (Fig. S5C). These results indicated that Gag stability was decreased by SGK1-S422D via a mechanism requiring the lysosomal pathway.

P62 (also known as sequestosome 1) is an autophagy receptor that is involved in lysosomal-mediated degradation of incoming pathogens (55, 56). Therefore, we tested whether PFV Gag interacts with p62. The Co-IP analysis showed that p62 interacted with PFV Gag, but not with Tas (Fig. 8G). Immunofluorescence analysis in HeLa cells also showed that Gag partially co-localized with p62 and microtubule associated protein 1 light chain 3 alpha (LC3) (Fig. S6A, B). Therefore, we hypothesized that Gag is transported to the lysosomal pathway for degradation. Autophagosomes fuse with lysosomes to form autolysosomes and ultimately degrade substrate proteins, in which lysosomal associated membrane protein 1 (LAMP1) is a lysosomal marker. Therefore, we tested whether PFV Gag co-localizes with LAMP1. Immunofluorescence experiments showed that PFV Gag and LAMP1 were co-localized, and SGK1-S422D enhanced the co-localization of Gag and lysosomes (Fig. 8H to J). These results indicated that SGK1-S422D enhanced the degradation of PFV Gag through the autophagic-lysosomal pathway.

### SGK1 phosphorylates Gag on Ser271 and Ser515, which facilitates the degradation of Gag.

**FIG 9:**
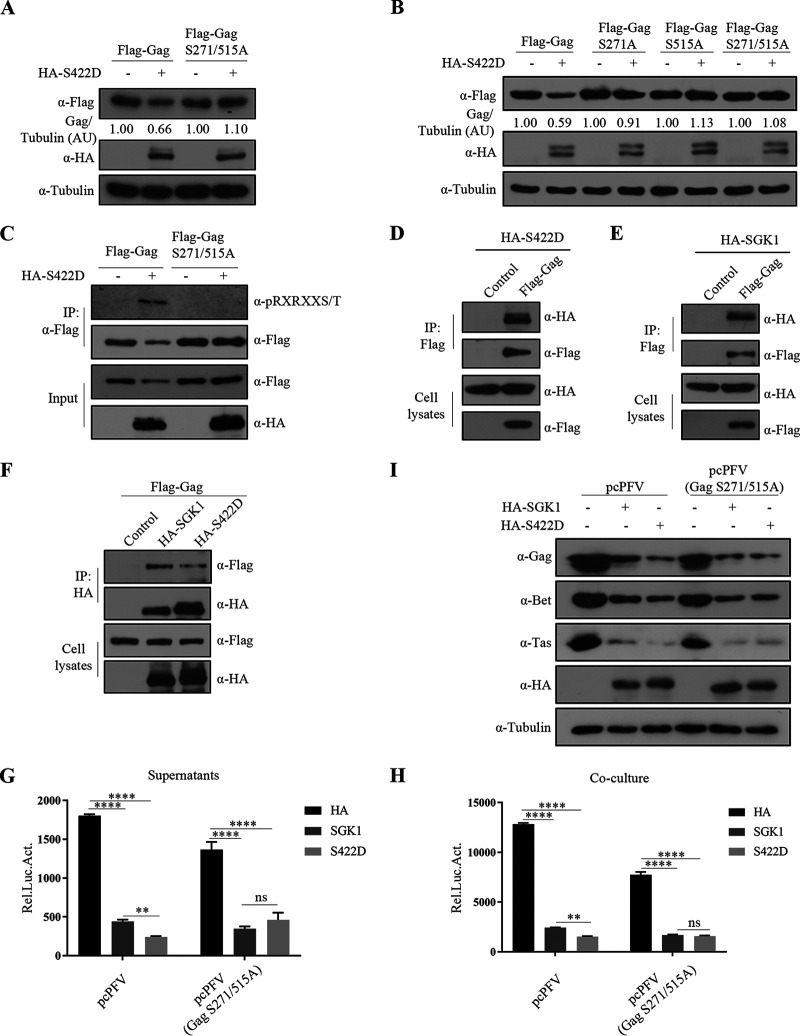
SGK1 phosphorylates Gag on Ser271 and Ser515, which facilitates the degradation of Gag. (A) HEK293T cells (2 × 10^5^) were transfected with Flag-Gag (0.6 μg), Flag-Gag S271/515A (0.6 μg), combined with SGK1-S422D or empty vector (0.3 μg). At 48 h posttransfection, cells were harvested for Western blotting analysis. Gag expression levels were quantified by densitometry and normalized to Tubulin protein levels. (B) HEK293T cells (2 × 10^5^) were transfected with Flag-Gag (0.6 μg), Flag-Gag S271A (0.6 μg), Flag-Gag S515A (0.6 μg), Flag-Gag S271/515A (0.6 μg), combined with SGK1-S422D or empty vector (0.3 μg). At 48 h posttransfection, cells were harvested for Western blotting analysis. Expression levels of Gag were quantified by densitometry and normalized to Tubulin protein levels. AU, arbitrary units. (C) HEK293T cells (4 × 10^6^) were transfected with Flag-Gag (5 μg), Flag-Gag S271/515A (5 μg), combined with SGK1-S422D or empty vector (5 μg). At 48 h posttransfection, co-immunoprecipitation was performed using Flag antibodies. Western blotting of samples from cell lysates and immunoprecipitates using HA, Flag and phospho (p)-AKT substrate antibodies. (D, E) HEK293T cells (4 × 10^6^) were transfected with HA-S422D (3 μg) (D) or HA-SGK1 (3 μg) (E) and empty vector or Flag-Gag (6 μg). At 48 h posttransfection, co-immunoprecipitation was performed using Flag antibodies. Western blotting of samples from cell lysates and immunoprecipitates using HA and Flag antibodies. (F) HEK293T cells (4 × 10^6^) were transfected with Flag-Gag (6 μg), combined with empty vector (3 μg), HA-SGK1 (3 μg), HA-S422D (3 μg). At 48 h posttransfection, co-immunoprecipitation was performed using HA antibodies. Western blotting of samples from cell lysates and immunoprecipitates using HA and Flag antibodies. (G to I) HEK293T cells (2 × 10^5^) were transfected with pcPFV or pcPFV (Gag S271/515A) (0.8 μg), combined with empty vector, SGK1 and SGK1-S422D (0.3 μg). At 30 h posttransfection, 600 μL of the supernatants (G) or 1/20 transfected cells (H) were incubated with PFVL cells (1 × 10^5^), the luciferase activity was measured 48 h later. (I) The rest of transfected cells were lysed for Western blotting. Data are expressed as the means ± standard deviations. Data are representative of three independent experiments. One-way ANOVA was used to perform the statistical test. **, *P < *0.01; ****, *P < *0.0001; ns for *P > *0.05.

SGK1 phosphorylation of its substrate protein leads to substrate degradation through the proteasomal and lysosomal pathways (52). SGK1 preferentially phosphorylates serine and threonine residues within an RXRXX(S/T) motif. Sequence analysis showed that Gag harbored the conserved serine residues, Ser271 and Ser515. We constructed a plasmid expressing the Gag S271/515A mutant and found that Gag S271/515A was resistant to SGK1-S422D-induced degradation (Fig. 9A). Next, to determine which serine residue of Gag is the SGK1-S422D trigger site, we constructed plasmids expressing the Gag S271A and Gag S515A mutants, respectively. Like Gag S271/515A, both Gag S271A and Gag S515A were resistant to degradation induced by SGK1-S422D; thus, SGK1-S422D acts on both the Ser271 and Ser515 residues of Gag (Fig. 9B).

We then investigated whether SGK1-S422D could phosphorylate Gag. A phospho-specific antibody recognizing the optimal (RXRXXS/T) phosphorylation consensus motif was used to detect Gag phosphorylation. The immunoprecipitation analysis revealed that even if SGK1-S422D reduced the Gag protein level, the level of Gag phosphorylation by SGK1-S422D increased, while SGK1-S422D could not reduce the amounts of Gag S271/515A protein, and no phosphorylation was detected (Fig. 9C). Examination of the interaction between SGK1, SGK1-S422D, and Gag showed that both proteins could interact with Gag (Fig. 9D to F). Thus, SGK1-S422D interacts with PFV Gag to enhance the phosphorylation of its Ser271 and Ser515 residues, which promotes PFV Gag degradation through the lysosome pathway. To verify the effect of SGK1-S422D on the stability of the Gag protein at the virus level, we constructed the pcPFV (Gag S271/515A) mutant plasmid. The results showed that the inhibitory effect of SGK1-S422D on wild-type PFV was stronger than SGK1; however, the inhibitory effect of SGK1-S422D on mutant PFV was equivalent to that of SGK1 (Fig. 9G to I). Thus, SGK1-S422D affects the stability of the Gag protein at the virus level.

## DISCUSSION

SGK1 plays an important role in many physiological and pathophysiological processes. Recently, studies found that SGK1 has an important role in virus infection. In this study, we report that PFV infection upregulates the promoter activity of *SGK1* via the Tas protein, which then upregulates SGK1 mRNA and protein levels. PFV Tas can upregulate the expression of certain cellular genes (57), and our findings enrich the understandings of this pathway. SGK1 inhibits the replication of PFV in two ways. First, SGK1 interacts with the Tas activation domain to inhibit Tas-induced transactivation. Second, constitutively active SGK1-S422 mediates Gag phosphorylation on Ser271 and Ser515, thereby increasing the degradation of Gag via lysosomal pathways.

Human SGK1 is very similar to bovine SGK1, and we demonstrated that both proteins inhibited the replication of PFV or BFV, although heterologous SGK1 inhibited the virus more significantly. This suggested that SGK1 might inhibit FVs from different species. SGK1 affects viral replication by interacting with Tas; however, there was no difference in the binding affinity of hSGK1 or bSGK1 to Tas and BTas (Fig. S7A, B). This type of species-independence is distinct from some retroviral restriction factors, such as Trim5α, which inhibits replication of SFVs from other species, but not their own (58), or Mx2, which is present in multiple primate species that share the capacity to potently inhibit HIV-1, whereas selected non-primate orthologs have no such activity (59). However, Nmi, APOBEC3G, and Tetherin may also be able to restrict SFVs from different species (17, 60).

The mechanism by which SGK1 affects the replication of certain viruses was unclear. The results of the present study showed that SGK1 does not affect PFV integration, which is consistent with the study of Rato et al., who found that SGK1 does not affect HIV integration (38). Tas acts as a switch for PFV from latent to lytic infection, and is very important for virus replication (61). Some cell factors affect the function of Tas through different mechanisms, thus inhibiting viral replication (13, 17, 20, 22). For instance, Pirh2 affects the Tas transactivation function by interacting with Tas and downregulating its expression (20); Nmi interacts with and diminishes the transactivation function of Tas by sequestrating it in the cytoplasm (17); PML inhibits PFV transcription by interacting with Tas to prevent its direct binding with viral DNA (13); and IFP35 inhibits the transcription of BFV by interacting with BTas to affect its recruitment of transcription factors (22). Herein, SGK1 was shown to interact with the Tas activation domain to affect Tas-induced transactivation, thus affecting viral transcription. Moreover, this inhibition is not cell type specific, possibly because Tas interacts with highly conserved component(s) of the cellular transcriptional machinery (49).

SGK1 negatively regulates certain transcription factors in different ways (53, 54, 62). For example, SGK1 downregulates p53 and Notch1 expression, which affects their transcriptional activity (53, 54) and inhibits FOXO3a-mediated transcription via nuclear exclusion (62). In contrast to the known effects of SGK1 on transcription factors, our results suggest that SGK1 does not affect the level and cellular localization of Tas, but affects its transcriptional activation function via interacting with its activation domain, similar to the effect of IFP35 on BTas (22). This is the first report that SGK1 acts an inhibitor of transcription factors in this way.

Phosphorylation regulates target protein recognition through ubiquitin-conjugating enzymes, thereby regulating protein stability (63). SGK1 also affects the stability of certain cellular proteins through lysosomal or proteasomal pathways in a kinase-dependent manner (52–54). For instance, SGK1 can phosphorylate Nicastrin, causing its degradation through lysosomal and proteasomal pathways (52). SGK1 kinase activity is important in Notch1 and p53 proteasomal degradation (53, 54). Other cellular proteins are phosphorylated and degraded, such as PKG1-mediated phosphorylation of Stathmin, which promotes its autophagic degradation (64). Herein, we showed that SGK1’s kinase activity mediated PFV Gag Ser271 and Ser515 phosphorylation, resulting in increased Gag degradation via the lysosomal pathway rather than proteasomal pathways. Comparison of wild-type virions with those containing Gag with RXRXXS mutations suggested that this motif plays a significant role in the response to SGK1 kinase-promoted Gag degradation at the virus level. We also showed that when Gag was expressed alone, its ubiquitination was not detected (Fig. S8A); whereas, when Gag was co-expressed with SGK1-S422D, Gag phosphorylation increased but its ubiquitination was still not detected (Fig. S8B). This is consistent with the study of Stanke et al., who also did not detect PFV Gag ubiquitination (65). Moreover, there is only one lysine in PFV Gag; however, SGK1-S422D still reduced the amount of the Gag K396R mutant protein (Fig. S8C), indicating Gag degradation after phosphorylation is not related to its ubiquitination. To the best of our knowledge, this is the first report to show that SGK1 phosphorylates viral proteins as a kinase. Further study of the molecular details of SGK1 kinase-mediated Gag degradation will provide clues regarding the selected host mechanism to degrade proteins.

The novel PFV inhibitor SGK1 functions by degrading Gag via the autophagy-dependent lysosomal pathway, and negatively regulates viral replication. The autophagolysosomal pathway is considered one of the oldest defenses against invading pathogens. Studies have described its antiviral effects through different mechanisms, including direct degradation of cytoplasmic viral components (66–71). For example, the chikungunya virus and the Sindbis virus capsid proteins are targeted for autophagic degradation (67–69); Tat in CD4^+^ T lymphocytes can be selectively degraded by autophagy (66); HIV-1 precursor protein gpl60 is degraded within lysosomes (70); and human T-cell leukemia virus type 1 Tax protein is a substrate for autophagy degradation (71). Additionally, autophagy negatively regulates the replication of PFV by an as-yet-unknown mechanism (72). Our results revealed a molecular mechanism involving the lysosomal pathway in PFV Gag degradation, thus regulating PFV replication. This result adds more evidence for the antiviral effect of autophagy and extends previous observations of the involvement of lysosomal pathways in PFV replication.

Phosphorylation of viral proteins significantly affects virus replication in host cells (73–76). For example, phosphorylation of Influenza NS1 reduces its binding to viral RNA, thereby negatively affecting viral replication (76). Phosphorylation of the Rubella capsid negatively regulates its viral genomic RNA binding activity, thereby affecting viral replication and assembly (75). Phosphorylation of the hepatitis B virus (HBV) core protein decreased viral DNA synthesis (74). PFV Gag is phosphorylated on multiple serine residues (77). Before the current work, the only kinase reported to mediate PFV Gag phosphorylation was polo-like kinase (PLK), which phosphorylates the S(T)P motif of PFV Gag and is important for the early replication steps of PFV; PLK kinase activity is required for the Gag-PLK interaction (78). Herein, we found that PFV Gag contains two consensus RXRXXS motifs which are recognition sites for the constitutively active form of SGK1, which acts on this motif to enhance Gag phosphorylation. The conservation of this motif suggests that SGK1 is another kinase of Gag; however, whether the phosphorylation of SGK1 is necessary for the observed protein-protein interaction requires further exploration.

**FIG 10:**
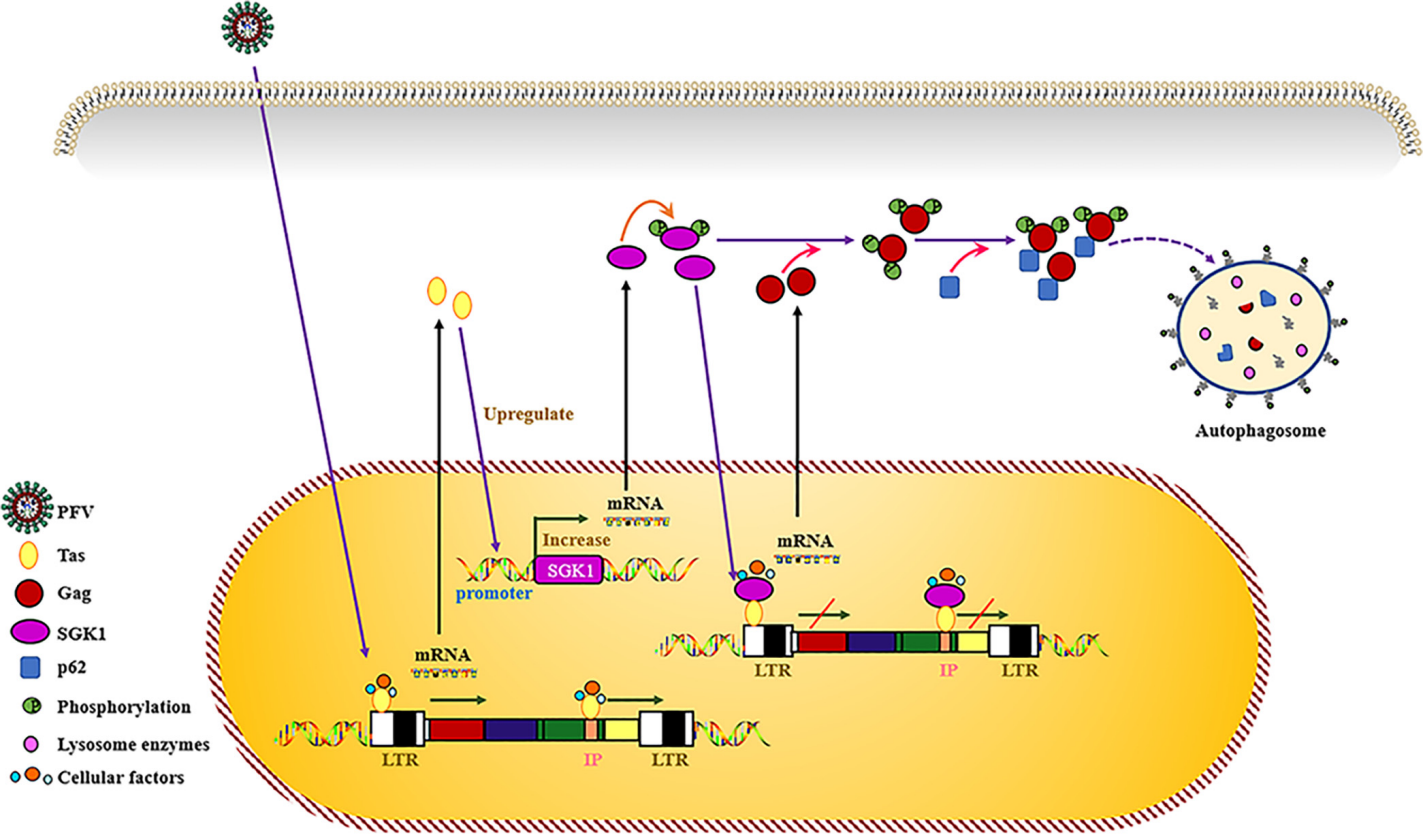
Schematic diagram of SGK1 inhibiting PFV replication. PFV infection enhances the promoter activity of *SGK1* through the Tas protein, and then upregulates the mRNA and protein levels of SGK1. On the one hand, SGK1 interacts with the Tas activation domain to affect its transcription activation function. On the other hand, the constitutively active form of SGK1 enhances the phosphorylation of PFV Gag which promotes Gag degradation via the lysosomal pathway.

In summary, we identified SGK1 as a novel inhibitor of PFV replication. As summarized in Fig. 10, PFV infection enhances the promoter activity of *SGK1* through Tas, thereby upregulating SGK1 mRNA and protein levels. SGK1 affects PFV replication mainly by altering PFV transcription, and can inhibit PFV to a lesser extent by reducing Gag protein stability. This not only furthers our understanding of SGK1’s kinase activity and identifies a new kinase acting on PFV Gag, but also complements the selective degradation of viral proteins by the autophagy pathway. The species-independent inhibition of FVs by SGK1 indicates that this might be a general mechanism to inhibit FVs replication and might contribute to the development of FVs as gene therapy vectors.

## MATERIALS AND METHODS

### Plasmids.

Human *SGK1* cDNA and bovine *SGK1* cDNA were cloned into the pCMV-3HA vector (Clonetech, Mountain View, CA, USA). SGK1-S222D and SGK1-K127N were generated using site-directed mutagenesis (Toyobo, Osaka, Japan) according to the manufacturer’s recommendations based on pCMV-3HA-SGK1. pGL-3262 was constructed based on a previous study (79). Plasmid DNA pLTR-Luc (80), pIP-Luc (80), and pcPFV (PFV full-length infectious clone) (81) were kindly provided by Maxine L. Linial (Division of Basic Sciences, Fred Hutchinson Cancer Research Center, Seattle, WA, USA). The p3.1-Tas, pFlag-Env, pFlag-Gag, pFlag-Tas, and pFlag-Bet DNA constructs were described previously (82). Constructs of the BFV infectious clone pBS-BFV were described previously (83). The pFlag-Gag S271A, S515A, S271/515A, and pcPFV (Gag S271/515A) were also generated using site-directed mutagenesis. All the mutations in this study were verified by DNA sequencing (Genewiz, Beijing, China).

### Cell culture and transfection.

HEK293T, HT1080, HeLa, PFVL, and BFVL cells were maintained in Dulbecco’s modified Eagle’s medium (DMEM) (Thermo Fisher Scientific, Waltham, MA, USA) supplemented with 10% fetal bovine serum (FBS) (HyClone, Logan, UT, USA) and 1% penicillin/streptomycin (Gibco, Grand Island, NY, USA). All cells were maintained at 37°C with 5% CO_2_. Plasmid transfections were performed using polyethylenimine (PEI, Polysciences, Warrington, PA, USA) (80) or Lipofectamine 3000 (Invitrogen, Carlsbad, CA, USA) in accordance with the manufacturer’s instructions.

### Reagents and antibodies.

DMSO, MG132, and Chloroquine were obtained from Sigma-Aldrich (St. Louis, MO, USA). Anti-Flag (M2), anti-HA, and anti-Myc antibodies were obtained from Santa Cruz Biotechnology (Santa Cruz, CA, USA). Anti-Tubulin antibodies and Protein A beads were obtained from Sigma-Aldrich. Antibodies against SGK1 (AF3200) were purchased from R&D Systems (Minneapolis, MN, USA). Antibodies against Phospho-Akt Substrate (RXRXXS*/T*) (23C8D2) and horseradish peroxidase-conjugated secondary antibodies were purchased from Cell Signaling Technology (Danvers, MA, USA). Fluorescein-and rhodamine-conjugated secondary antibodies were purchased from Jackson Immuno Research Laboratories (West Grove, PA, USA). Polyclonal mouse sera against PFV Gag, Tas, or Bet were prepared as described previously (82). The antibodies to BFV-Gag and BTas were also produced as described previously (22, 84).

### Virus infections.

To prepare virus stocks, the PFV infectious clone pcPFV was transfected into HEK293T cells, and after 48 h, the supernatant was collected and filtered through a 0.45-μm pore-size filter membrane. The virus titer was determined by infecting PFVL cells as described previously (17), and then the multiplicity of infection (MOI) was calculated according to the method of Tai et al. (45). Cells were infected with the PFV stock. After 8 h, the virus inoculum was washed off and fresh medium was added. After another 40 h, the culture supernatants or infected cells were incubated with PFVL cells (1 × 10^5^) and the viral load was measured via the luciferase activity.

### Generation of stably transduced cell lines.

To screen cell lines stably expressing *SGK1*, HEK293T cells were transfected with 1 μg of pMLV -Gag-Pol, 0.5 μg of pVSV-G, and 1 μg of pQC-SGK1. After 48 h, the supernatants were harvested to infect HT1080 cells. Forty-eight hours after infection, cells were subcultured in selection medium containing 2 μg/mL puromycin (Sigma-Aldrich,). Western blotting was used to analyze the efficiency of SGK1 expression.

The *SGK1* knockdown HT1080 cell line was screened using sgRNA. HEK293T cells were transfected with 1 μg of psPAX2, 0.5 μg of pVSV-G, and 1 μg of pLentiCRISPR (with sgRNA cloned). After 48 h, the supernatants were harvested to infect HT1080 cells. Forty-eight hours after infection, cells were subcultured in selection medium containing 2 μg/mL puromycin. Western blotting was used to analyze the efficiency of *SGK1* knockdown. The target sequence for the *SGK1* sgRNAs were as follows: sgRNA1#: forward (5′-CACCGCCTCATCCTGGAGTAAGTGA-3′); reverse (5′-AAACTCACTTACTCCAGGATGAGGC-3′); sgRNA2#: forward (5′-CACCG GGGTTGGCATTCATAAGCTC-3′); reverse (5′-AAACGAGCTTATGAATGCCAA CCCC-3′); sgRNA3#: forward (5′-CACCGCCTTCTCAGCAAATCAACCT-3′); reverse (5′-AAACAGG TTGATTTGCTGAGAAGGC-3′).

### Luciferase reporter assay.

Cells were harvested 48 h after infection or transfection and luciferase activity was measured using a luciferase reporter assay system kit (Promega, Madison, WI, USA) according to the manufacturer’s protocol.

### Alu-PCR.

To detect the integration level of the virus, HT1080 cells stably expressing *SGK1* and control cells were inoculated in 12-well plates. The experimental group was treated with reverse transcriptase inhibitor AZT (10 μM) (85) and integrase inhibitor Raltegravir (10 μM) (86) 2 h before virus infection. Two hours later, PFV stock was added to infect the cells, and 48 h after infection, the cells were harvested and total DNA was extracted using a DNeasy blood and tissue kit according to the manufacturer’s instructions (Qiagen, Hilden, Germany). According to the principle of Alu-PCR (87), semi-quantitative PCR and real-time PCR were used to detect the integrated PFV genome. The Alu-PCR primers were as follows: ALU1 TCCCAGCTACTGGGGAGGCTGAGG, ALU2 GCCTCCCAAAGTGCTGGGATTA CAG, SpA ATGCCACGTAAGCGAAACTTAGTATAATCATTTCCGCTTTCG, Lambda ATGCCACGTAAGCAAACT, NestedR GAAACTAGGGAAAACTAGG.

### Co-immunoprecipitation.

To analyze protein interactions, HEK293T cells were transfected with corresponding plasmids. After 48 h, the cells were harvested and lysed in immunoprecipitation buffer (50 mM Tris, 150 mM NaCl, 2 mM EDTA, 3% Glycerol, 1% Triton X-100, EDTA-free protease inhibitor cocktail tablets). After brief sonication, cell lysates were centrifuged at 4°C and 12,000 × *g* for 15 min. The supernatant was incubated with antibodies at 4°C for 3 h, and then incubated with rotation with Protein A-agarose. After 3 h, the immunoprecipitated materials were washed six times with lysis buffer, and then boiled with 2 × SDS loading buffer and analyzed using Western blotting.

### Cross-linking assay.

The cross-linking assay was performed as previously described (22). HA-SGK1 or empty vector was co-transfected with Flag-Tas into HEK293T cells. Forty-eight hours later, cells were lysed using cross-linking lysis buffer and sonicated briefly. After centrifugation at 4°C and 12,000 × *g* for 10 min, the supernatant was incubated with cross-linking buffer at room temperature for 30 min. SDS-PAGE loading buffer was added to terminate the cross-linking reaction. Proteins were subjected to Western blotting using the indicated antibodies.

### Western blotting.

Transfected or infected cells were harvested and lysed in radioimmunoprecipitation assay (RIPA) buffer at 4°C for 30 min. The protein concentration was quantified using a BCA protein assay (Beyotime Biotechnology, Shanghai, PRC). Then, the protein lysates were centrifuged at 13,000 × *g* for 15 min at 4°C to remove the precipitate. The supernatants were collected and added appropriate amount of 6 × SDS loading buffer, and boiled at 100°C for 15 min. Samples were run on SDS-PAGE and transferred to PVDF membranes (GE Healthcare, Chicago, IL, USA). The membranes were blocked with 5% nonfat milk at room temperature for 45 min, and then incubated with primary antibodies and secondary antibodies at room temperature for 1.5 h and 45 min, respectively. After that, horseradish peroxidase substrate was added to the membrane and used for signal detection.

### Chromatin immunoprecipitation assay.

The chromatin immunoprecipitation (ChIP) assay was performed in PFVL cells or HEK293T cells. ChIP analysis was conducted using an EZ-chip kit (Millipore, Burlington, MA, USA) according to the manufacturer’s instructions. Cells were transfected with corresponding plasmids. After 48 h, formaldehyde was added to the culture medium at a final concentration of 1% and kept at 37°C for 10 min. After sonication, the chromatin was immunoprecipitated using specific antibodies or control antibodies. DNA was purified and subjected to PCR to detect PFV LTR or IP DNA using the primer pair LTR forward (5′-GTGAGATCGAATCTTTCCTTAAC-3′) and LTR reverse (5′-CCGTA CAATCTAGAAACTATCC-3′); IP forward (5′-CGTGACTGTTAATGAAACAA CG-3′) and IP reverse (5′-GCTTTTGCTCTTTCAATCTGCTC-3′). *GAPDH* promoter specific DNA using the primer pair forward (5′-TACTAGCGGTTTTACGGGCG-3′) and reverse (5′-TCGAACAGGAGGAGCAGAGAGCGA-3′) was also amplified from a ChIP assay and used as a control.

### Data availability.

The sequencing data were deposited at the Gene Expression Omnibus (GEO) repository (accession number is GSE200199).

### Statistical analysis.

Statistical analysis was performed using the GraphPad Prism software (GraphPad Inc. La Jolla, CA, USA). On the figures, *P*-values are indicated as follows: *, *P < *0.05; **, *P < *0.01; ***, *P < *0.001; ****, *P < *0.0001; and ns for *P > *0.05.
