# Supplementary material for: SGK1, a Serine/Threonine Kinase, Inhibits Prototype Foamy Virus Replication
Source: Microbiol Spectr. 2022 Apr 19;10(3):e01995-21. doi: 10.1128/spectrum.01995-21 (PMC9241813; doi:10.1128/spectrum.01995-21)
Supplement: SUPPLEMENTAL FILE 5 — Figure S1-8. Download spectrum.01995-21-s005.pdf, PDF file, 0.9 MB [file spectrum.01995-21-s005.pdf]

A

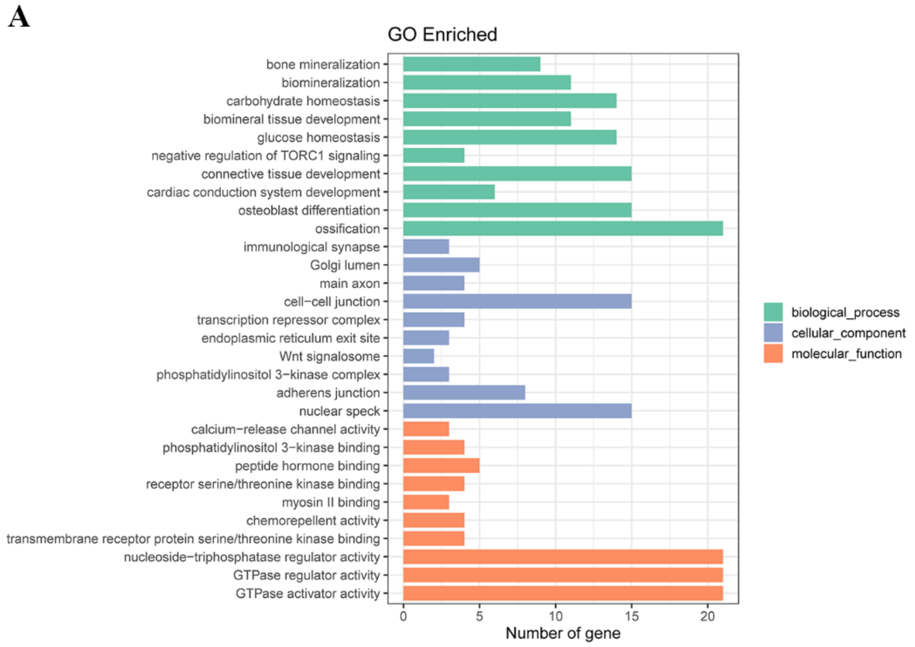

B

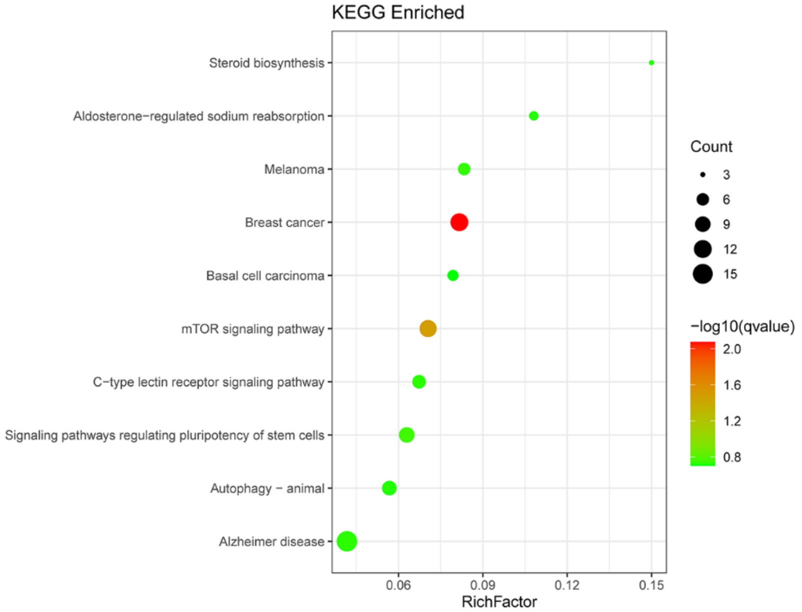

C

Description	Gene
mTOR signaling pathway	*LPIN3, WNT5B, PIK3R1, FZD1, DEPTOR, SGK1, PIK3CD, WNT3, RNF152, ATP6V1E2, IGF1R*

Fig S1 Top 10 significant enrichment GO and KEGG terms of DEGs. (**A**) GO enrichment analysis of differential genes. (**B**) KEGG enrichment analysis of differential genes. (**C**) DEGs in the mTOR signaling pathway.

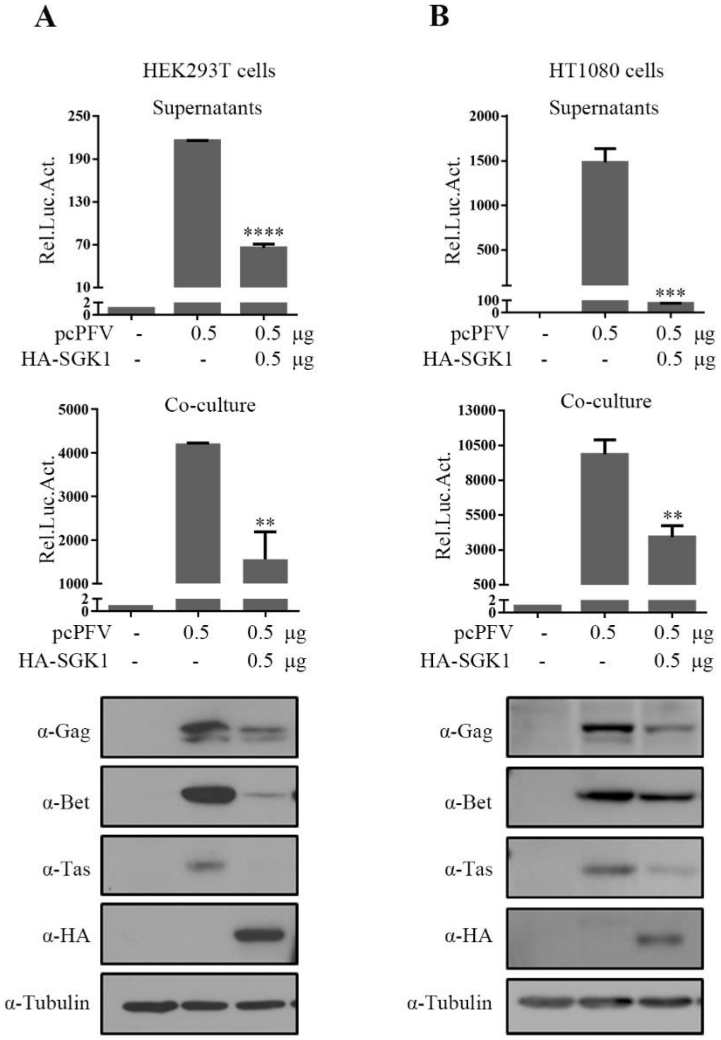

Fig S2 Overexpression of SGK1 inhibits PFV replication. (**A**) HEK293T cells (2×10^5) and (**B**) HT1080 cells (1×10^5) were co-transfected with pcPFV (0.5 μg) and empty vector or SGK1 (0.5 μg). At 48 h post-transfection, 600 μl of the supernatants or 1/10 transfected cells were incubated with PFVL cells (1×10^5), and the luciferase activity was measured 48 h later. The remaining transfected cells were lysed for western blotting. Data are expressed as the means ± standard deviations. Data are representative of three independent experiments. One-way ANOVA was used to perform the statistical test. ** $p < 0.01$, *** $p < 0.001$, **** $p < 0.0001$.

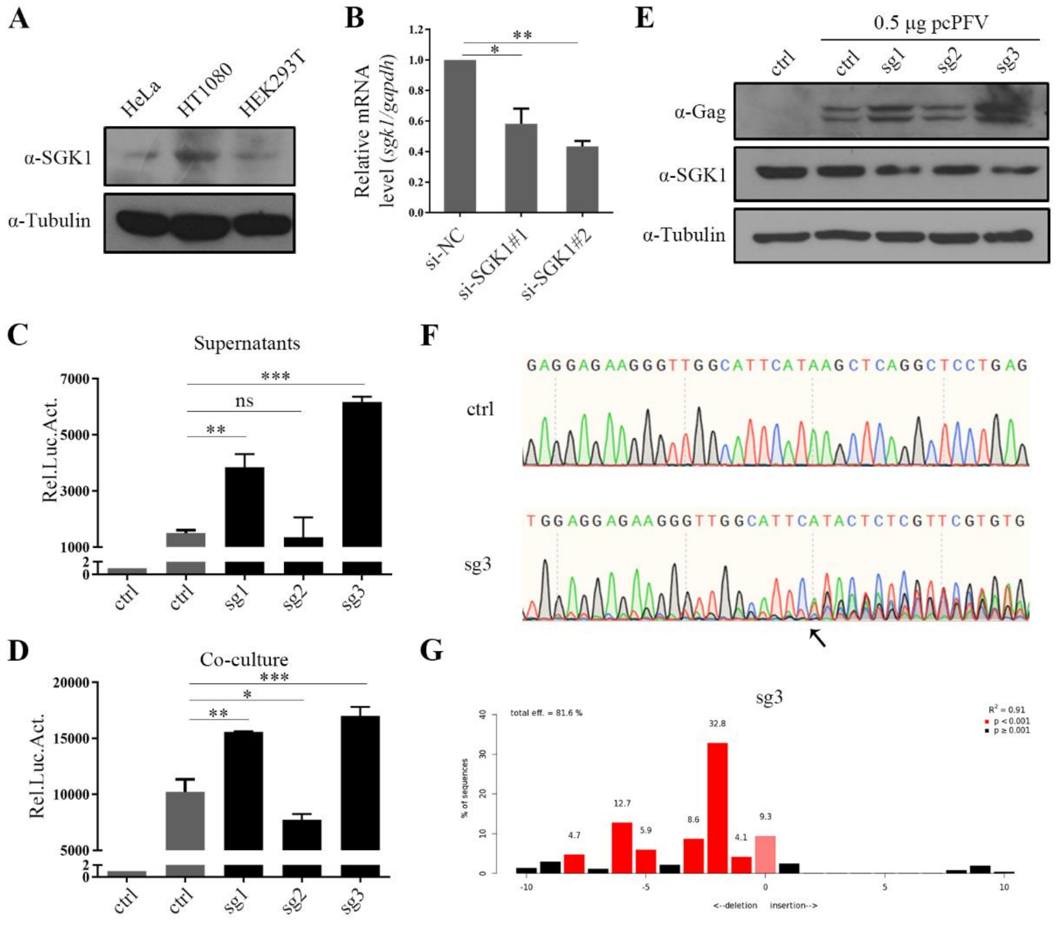

Fig S3 Knockdown of endogenous SGK1 enhances PFV replication. (**A**) HeLa, HT1080 and HEK293T cells (2×10^6) were lysed for western blotting. (**B**) The relative level of SGK1 mRNA was quantified by RT-PCR. (**C–E**) Control, sg1, sg2 and sg3 cell lines (1×10^5) were transfected with pcPFV (0.5 μg). At 48 h post-transfection, 600 μl of the supernatants (**C**) or 1/10 transfected cells (**D**) were incubated with PFVL cells (1×10^5), the luciferase activity was measured 48 h later. (**E**) The rest of transfected cells were lysed for western blotting. (**F**) Sanger sequencing to detect indel spectrums of sg3. Black arrows indicate the cleavage site of CRISPR/Cas9. (**G**) TIDE analysis of the knockdown efficiencies. Data are expressed as the means ± standard deviations. Data are representative of two independent experiments. One-way ANOVA was used to perform the statistical test. * $p < 0.05$, ** $p < 0.01$, *** $p < 0.001$ and ns for $p > 0.05$.

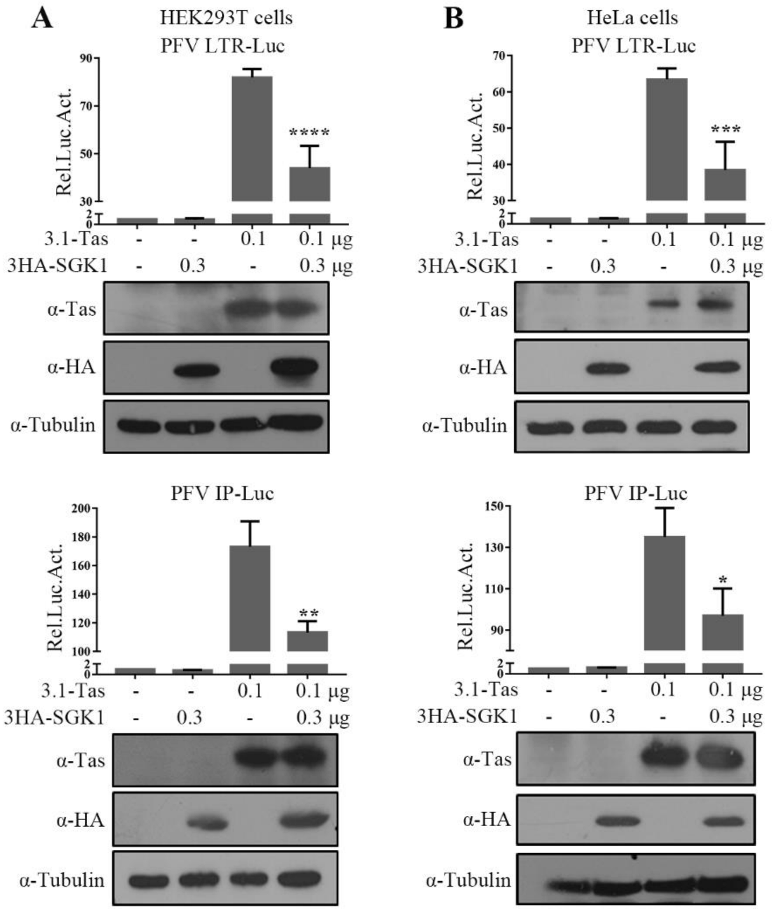

Fig S4 SGK1 inhibits Tas from transactivating PFV LTR and IP promoters. HEK293T cells (2 × 10^5) (**A**) and HeLa cells (1 × 10^5) (**B**) were transfected with LTR-Luc (0.025 μg) or IP-Luc (0.01 μg), combined with 3.1-Tas and empty vector or SGK1. At the same time, pCMV-β-gal (0.025 μg) was transfected to normalize transfection efficiency. At 48 h post-transfection, luciferase activities were measured and corrected by β-gal catalytic activities. Remaining cell lysate for western blotting. Data are expressed as the means ± standard deviations. Data are representative of three independent experiments. One-way ANOVA was used to perform the statistical test. * $p < 0.05$, ** $p < 0.01$, *** $p < 0.001$ and **** $p < 0.0001$.

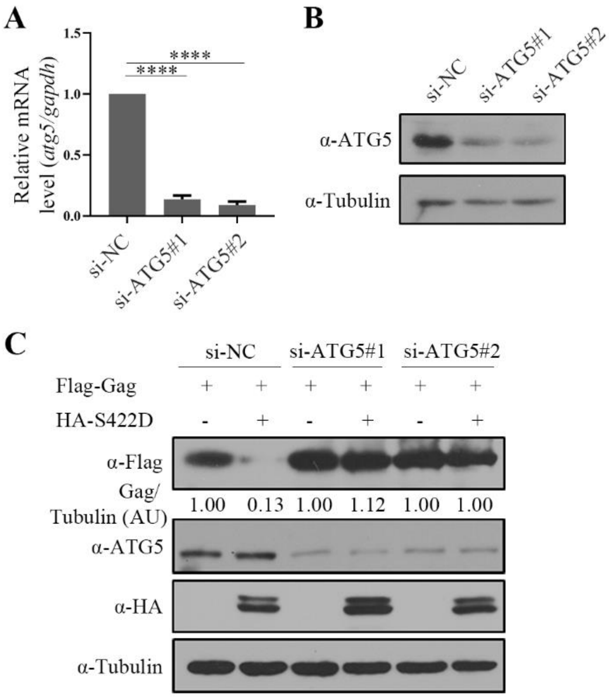

Fig S5 Knockdown of ATG5 prevents the degradation of Gag by S422D. (**A**, **B**) HEK293T cells (2×10^5) were transfected with siNC or siATG5 (30 pmol). At 30 h post-transfection, cells were harvested to detect ATG5 knockdown efficiency. (**A**) The relative level of ATG5 mRNA was quantified by RT-PCR. (**B**) The level of ATG5 protein was quantified by western blotting. (**C**) HEK293T cells (2×10^5) were transfected with siNC or siATG5. After 6 h, cells were co-transfected with Flag-Gag (0.6 μg) and S422D or empty vector (0.3 μg). After another 24 h, cells were collected for western blotting analysis. NC: negative control. **** $p < 0.0001$.

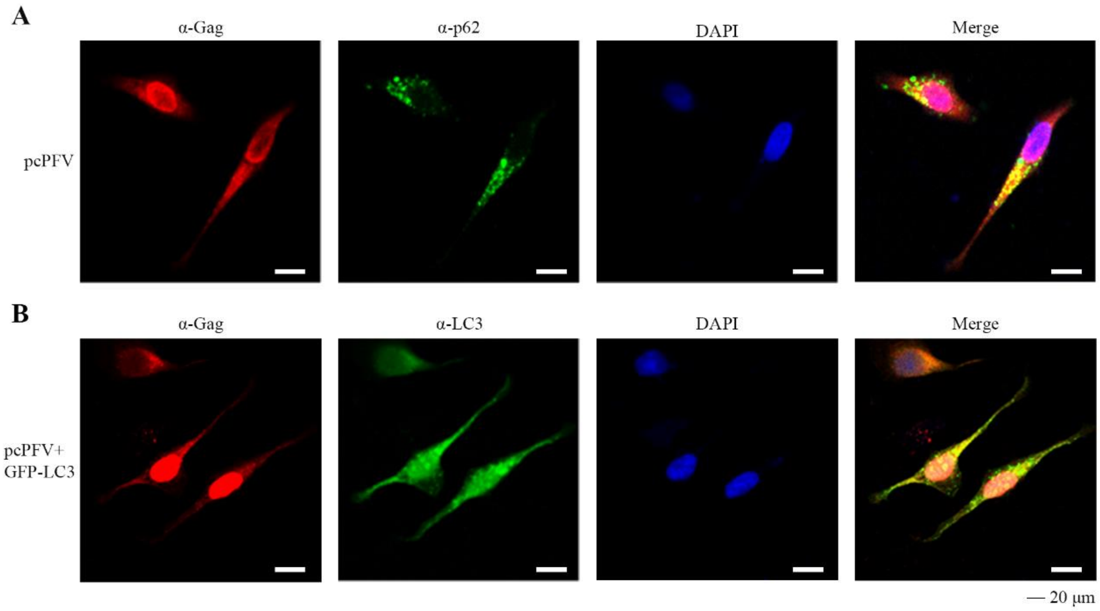

Fig S6 Gag co-localized with p62 and LC3. (**A**, **B**) HeLa cells (3 × 10^4) were transfected with pcPFV (0.8 μg) alone or pcPFV (0.8 μg) and GFP-LC3 (0.7 μg). At 48 h post-transfection, an indirect IFA was used to localize Gag (with Gag antibody and tetramethyl rhodamine isocyanate [TRITC]-conjugated secondary antibody) and p62 or LC3 (with p62 or LC3 antibodies and fluorescein isothiocyanate [FITC]-conjugated secondary antibody). Nuclei were visualized with DAPI staining. Representative images are shown. Scale bars, 20 μm.

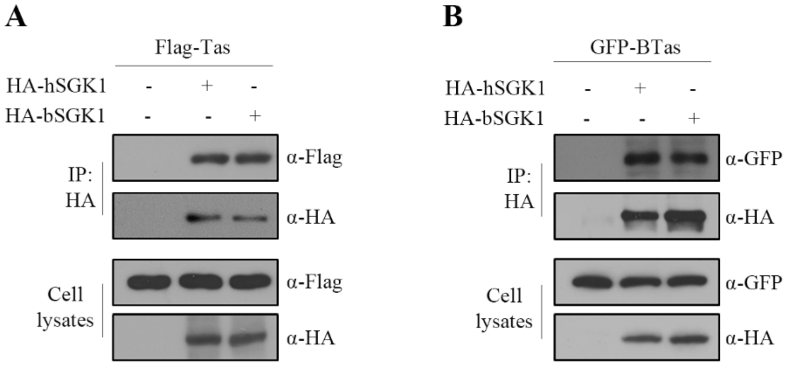

Fig S7 Both hSGK1 and bSGK1 interacted with Tas or BTas. (**A**, **B**) HEK293T cells (4×10^6) were transfected with 3 μg of Flag-Tas (**A**) or 3 ug of GFP-BTas (**B**), combined with empty vector (3 μg), HA-hSGK1 (3 μg) or HA-bSGK1 (3 μg). At 48 h post-transfection, co-immunoprecipitation was performed and western blotting analysis of samples from cell lysates.

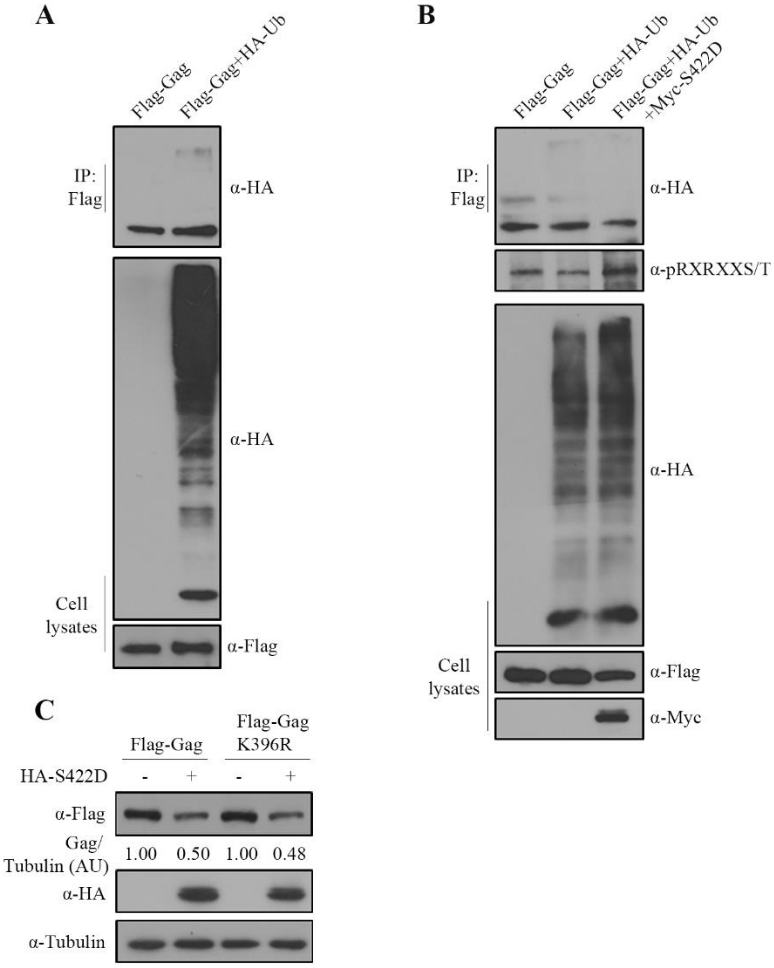

Fig S8 S422D enhances Gag degradation independent of ubiquitination. (**A**) HEK293T cells (4 × 10^6) were transfected with Flag-Gag (5 μg) and vector (3 μg) or HA-Ub (3 μg). At 48 h post-transfection, co-immunoprecipitation was performed and western blotting analysis of samples from cell lysates. (**B**) HEK293T cells (4 × 10^6) were transfected with Flag-Gag (5 μg) and empty vector, HA-Ub (3 μg) or HA-Ub (3 μg) +Myc-S422D (5 μg). At 48 h post-transfection, co-immunoprecipitation was performed and western blotting analysis of samples from cell lysates. (**C**) HEK293T cells (2 × 10^5) were transfected with Flag-Gag (0.6 μg), Flag-Gag K396R (0.6 μg), combined with S422D or empty vector (0.3 μg). At 48 h post-transfection, cells were harvested for western blotting analysis.
